# Supplementary material for: Neofunctionalization of Duplicated P450 Genes Drives the Evolution of Insecticide Resistance in the Brown Planthopper
Source: Curr Biol. 2018 Jan 22;28(2):268–274.e5. doi: 10.1016/j.cub.2017.11.060 (PMC5788746; doi:10.1016/j.cub.2017.11.060)
Supplement: Document S1. Figures S1–S4 and Tables S1–S3 [file mmc1.pdf]

Current Biology, Volume *28*

Supplemental Information

Neofunctionalization of Duplicated P450 Genes Drives the Evolution of Insecticide Resistance in the Brown Planthopper

Christoph T. Zimmer, William T. Garrood, Kumar Saurabh Singh, Emma Randall, Bettina Lueke, Oliver Gutbrod, Svend Matthiesen, Maxie Kohler, Ralf Nauen, T.G. Emyr Davies, and Chris Bass

A

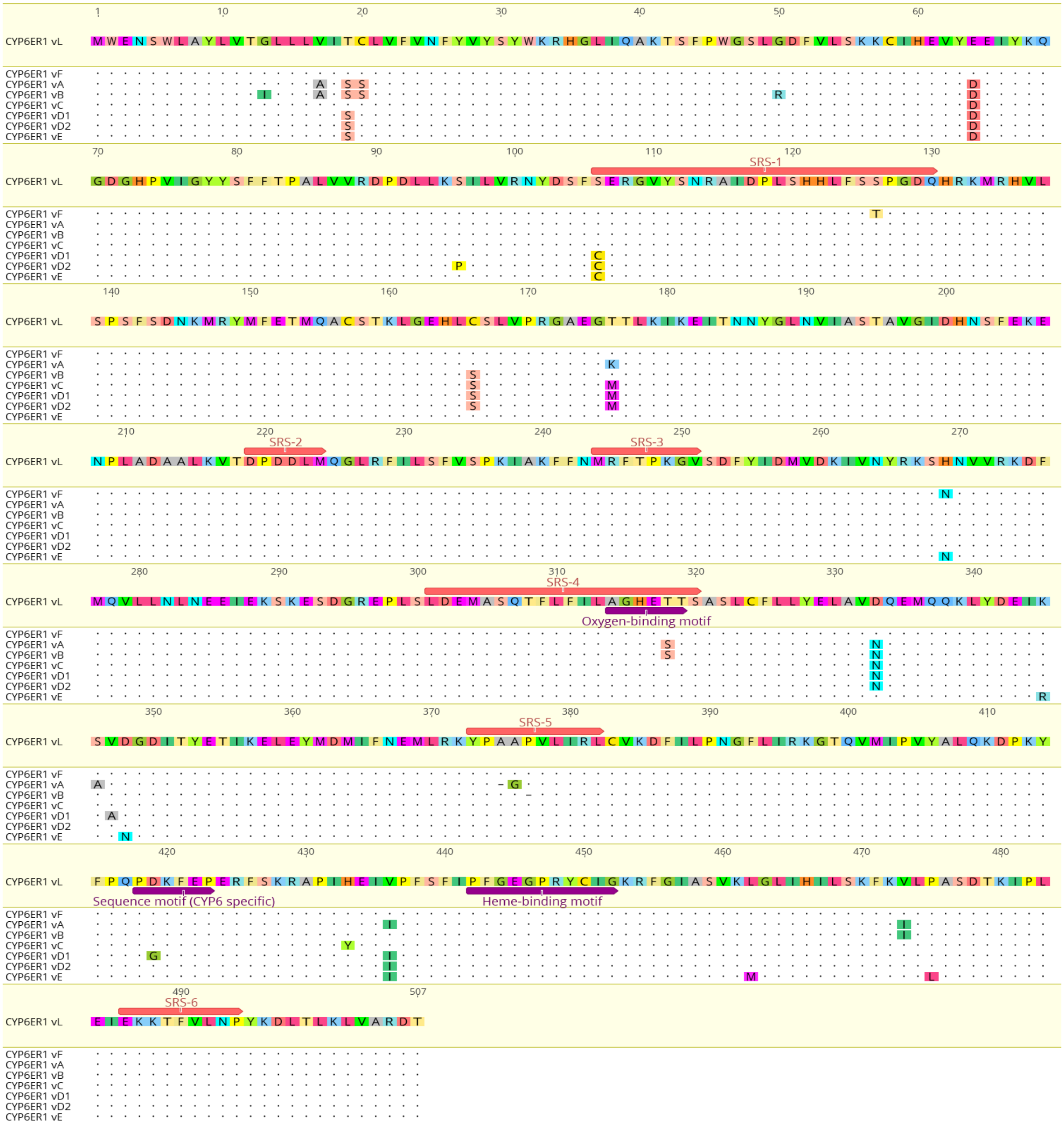
CYP6ER1 vL
CYP6ER1 vF
CYP6ER1 vA
CYP6ER1 vB
CYP6ER1 vC
CYP6ER1 vD1
CYP6ER1 vD2
CYP6ER1 vE
SRS-1
SRS-2
SRS-3
SRS-4
Oxygen-binding motif
SRS-5
Sequence motif (CYP6 specific)
Heme-binding motif
SRS-6
507

B

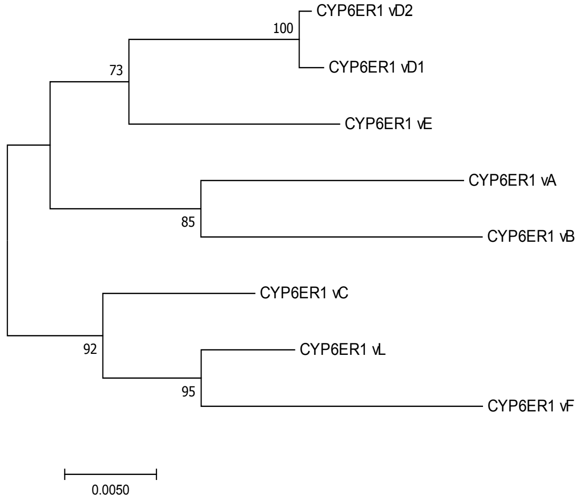
CYP6ER1 vD2
100
CYP6ER1 vD1
73
CYP6ER1 vE
CYP6ER1 vA
85
CYP6ER1 vB
CYP6ER1 vC
92
CYP6ER1 vL
95
CYP6ER1 vF
0.0050

Figure S1. Sequence characterisation of CYP6ER1 variants. Related to Figure 1.
A) Amino acid alignment of CYP6ER1 variants. Key conserved P450 motifs and substrate recognition sites are annotated. B) Phylogenetic relationship of CYP6ER1 variants. Tree was generated using the Maximum Likelihood method and is drawn to scale, with branch lengths measured in the number of substitutions per site. Bootstrap replications were performed and the percentage of 1000 replications supporting each branch are shown.

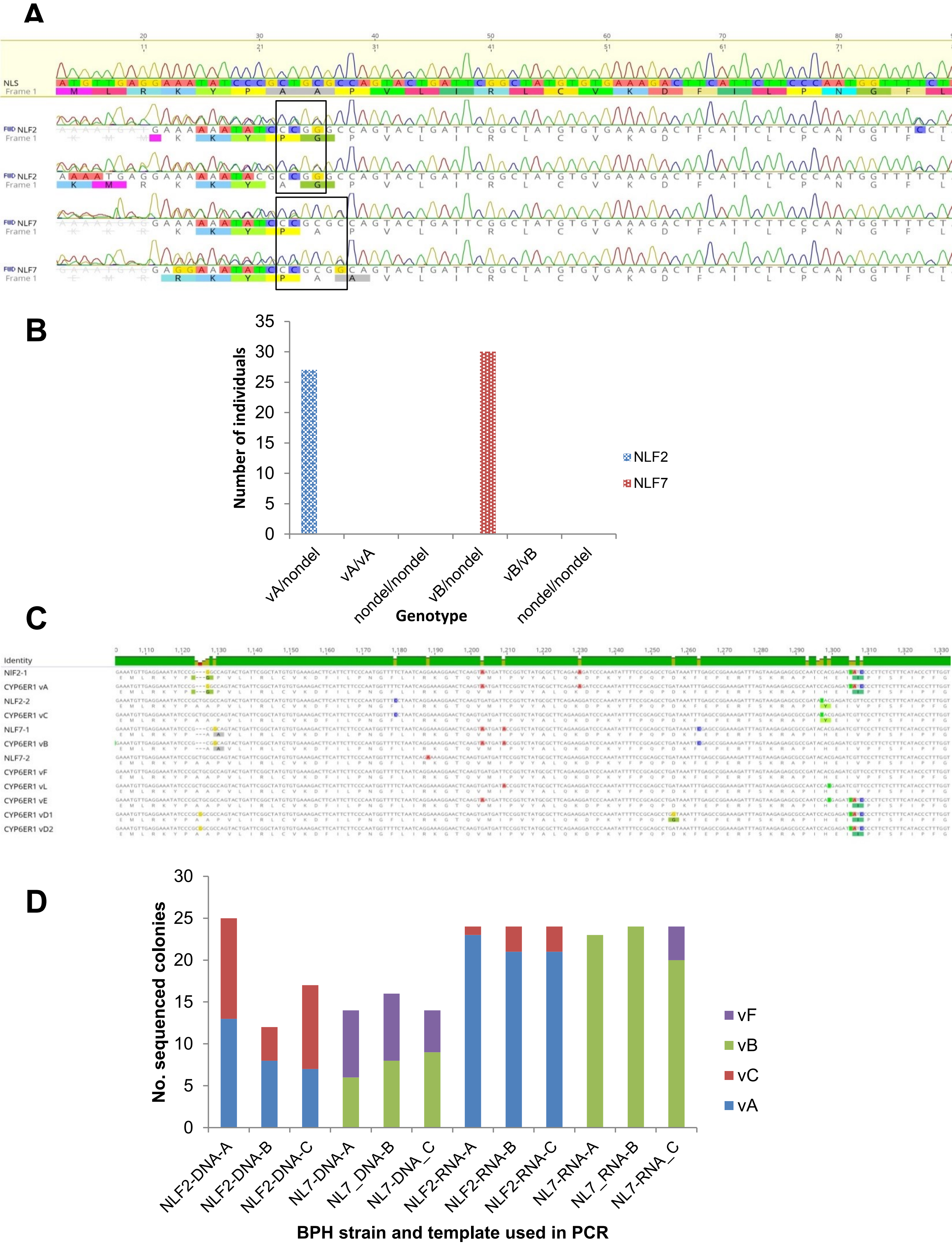
A
NLS
Frame 1
NLF2
NLF7
B
Number of individuals
Genotype
vA/nondel
vA/vA
nondel/nondel
vB/nondel
vB/vB
nondel/nondel
NLF2
NLF7
C
Identity
NIF2-1
CYP6ER1 vA
NLF2-2
CYP6ER1 vC
NLF7-1
CYP6ER1 vB
NLF7-2
CYP6ER1 vF
CYP6ER1 vL
CYP6ER1 vE
CYP6ER1 vD1
CYP6ER1 vD2
D
No. sequenced colonies
BPH strain and template used in PCR
NLF2-DNA-A
NLF2-DNA-B
NLF2-DNA-C
NL7-DNA-A
NL7_DNA-B
NL7-DNA_C
NLF2-RNA-A
NLF2-RNA-B
NLF2-RNA-C
NL7-RNA-A
NL7_RNA-B
NL7-RNA_C
vF
vB
vC
vA

Figure S2. *CYP6ER1* genotyping of individuals of the NLF2 and NLF7 BPH strains using DNA and RNA as template in (RT-)PCR. Related to Figure 3. (A) Representative sequence traces obtained from direct sequencing of a diagnostic fragment of *CYP6ER1* encompassing the resistance mutation sites in exon 6. DNA extracted from individuals of the NLF2 and NLF7 strains was used as template in PCR. Two representative sequences of each strain are aligned to a representative sequence obtained from NLS (which has just one copy of *CYP6ER1*). Boxed regions indicate the sites of the A375del+A376G mutations in *CYP6ER1v*A and P377del in *CYP6ER1vB* (see also part C of this figure) and highlight the heterozygosity observed at these regions (overlapping chromatogram peaks). (B) Results of genotyping 27-30 individuals of the NLF2 and NLF7 for the resistance mutations that define *CYP6ER1v*A and *CYP6ER1vB*. (C) Alignment of a diagnostic *CYP6ER1* sequence fragment from BPH individuals of resistant strains. Alignment shows representative sequence reads obtained from cloning and sequencing an amplicon containing SNPs and INDELs diagnostic for each of the unique *CYP6ER1* variants using DNA extracted from individuals of the NLF2 and NLF7 strains. For reference the sequence of the eight *CYP6ER1* variants is included. (D) Number of sequenced colonies obtained of each *CYP6ER1* variant after cloning and sequencing PCR products amplified from either genomic DNA or mRNA of individuals of the NLF2 and NLF7 strains.

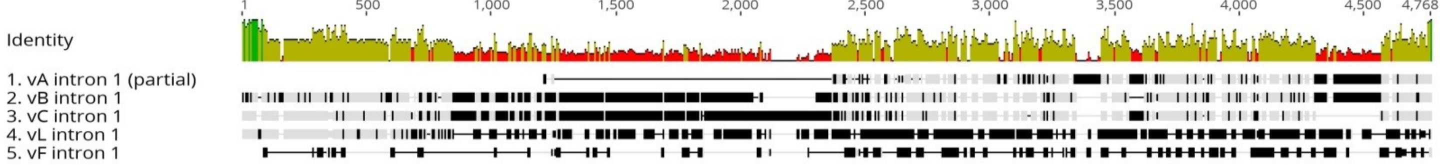

	vA intron 1 (...	vB intron 1	vC intron 1	vL intron 1	vF intron 1
vA intron 1 (partial)		62.788%	53.471%	29.240%	19.647%
vB intron 1	62.788%		83.395%	44.544%	15.689%
vC intron 1	53.471%	83.395%		44.869%	15.148%
vL intron 1	29.240%	44.544%	44.869%		18.416%
vF intron 1	19.647%	15.689%	15.148%	18.416%	

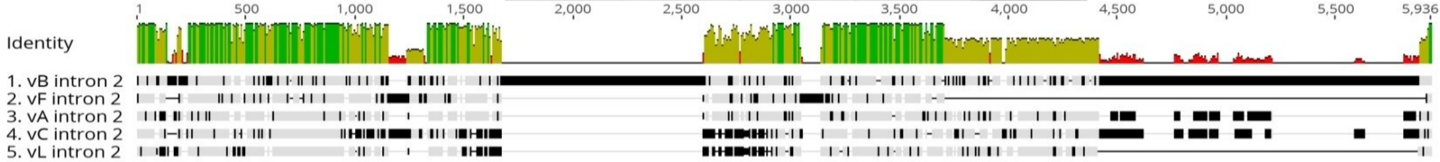

	vB intron 2	vF intron 2	vA intron 2	vC intron 2	vL intron 2
vB intron 2		40.450%	57.032%	53.548%	49.855%
vF intron 2	40.450%		59.553%	56.943%	62.544%
vA intron 2	57.032%	59.553%		74.154%	80.210%
vC intron 2	53.548%	56.943%	74.154%		75.513%
vL intron 2	49.855%	62.544%	80.210%	75.513%	

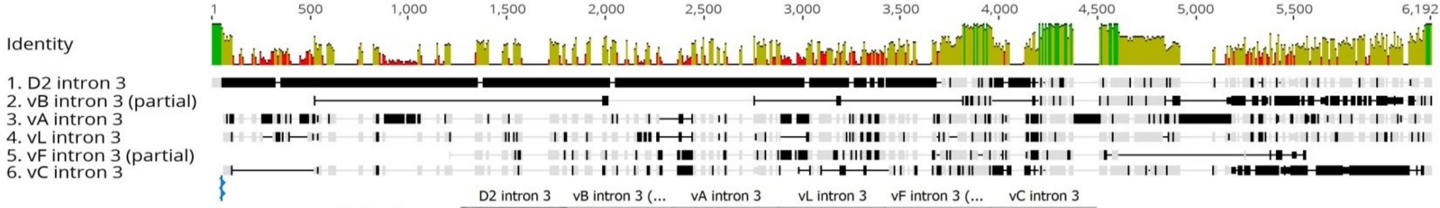

	D2 intron 3	vB intron 3 (...	vA intron 3	vL intron 3	vF intron 3 (...	vC intron 3
D2 intron 3		21.895%	43.938%	44.345%	32.276%	35.004%
vB intron 3 (partial)	21.895%		28.853%	34.383%	18.323%	30.205%
vA intron 3	43.938%	28.853%		78.910%	62.323%	55.845%
vL intron 3	44.345%	34.383%	78.910%		64.527%	63.801%
vF intron 3 (partial)	32.276%	18.323%	62.323%	64.527%		56.042%
vC intron 3	35.004%	30.205%	55.845%	63.801%	56.042%	

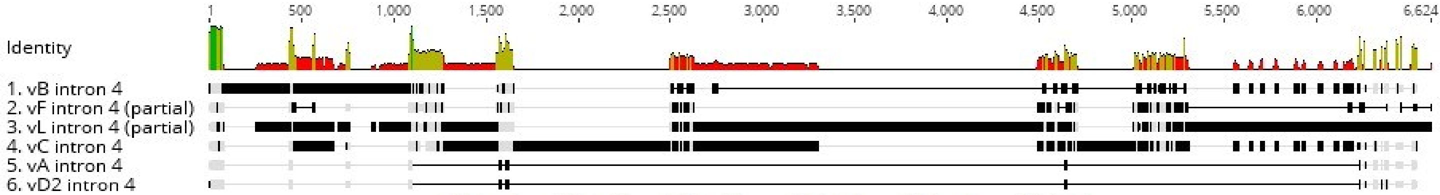

	vB intron 4	vF intron 4 (...	vL intron 4 (...	vC intron 4	vA intron 4	vD2 intron 4
vB intron 4		27.966%	26.264%	25.910%	12.677%	12.540%
vF intron 4 (partial)	27.966%		19.464%	27.521%	18.982%	18.809%
vL intron 4 (partial)	26.264%	19.464%		37.179%	5.025%	4.965%
vC intron 4	25.910%	27.521%	37.179%		7.872%	7.792%
vA intron 4	12.677%	18.982%	5.025%	7.872%		99.051%
vD2 intron 4	12.540%	18.809%	4.965%	7.792%	99.051%	

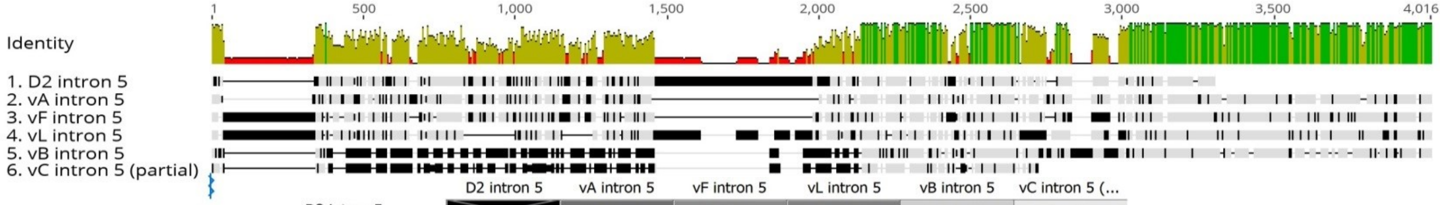

	D2 intron 5	vA intron 5	vF intron 5	vL intron 5	vB intron 5	vC intron 5 (...
D2 intron 5		72.941%	66.422%	68.793%	50.169%	43.824%
vA intron 5	72.941%		81.403%	69.075%	67.483%	54.206%
vF intron 5	66.422%	81.403%		76.612%	60.167%	46.750%
vL intron 5	68.793%	69.075%	76.612%		54.709%	38.457%
vB intron 5	50.169%	67.483%	60.167%	54.709%		91.983%
vC intron 5 (partial)	43.824%	54.206%	46.750%	38.457%	91.983%	

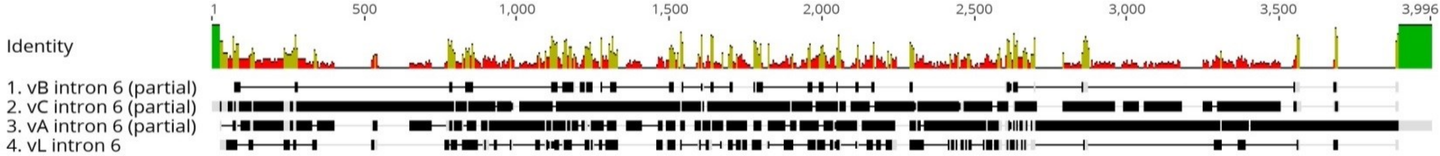

	vB intron 6 (...	vC intron 6 (...	vA intron 6 (...	vL intron 6
vB intron 6 (partial)		7.910%	8.061%	19.212%
vC intron 6 (partial)	7.910%		33.069%	19.281%
vA intron 6 (partial)	8.061%	33.069%		21.562%
vL intron 6	19.212%	19.281%	21.562%	

Figure S3. **Sequence analysis of the introns of different *CYP6ER1* variants. Related to Figure 3.**

Alignment of each intron is shown with a matrix of calculated sequence identity shown below.

A

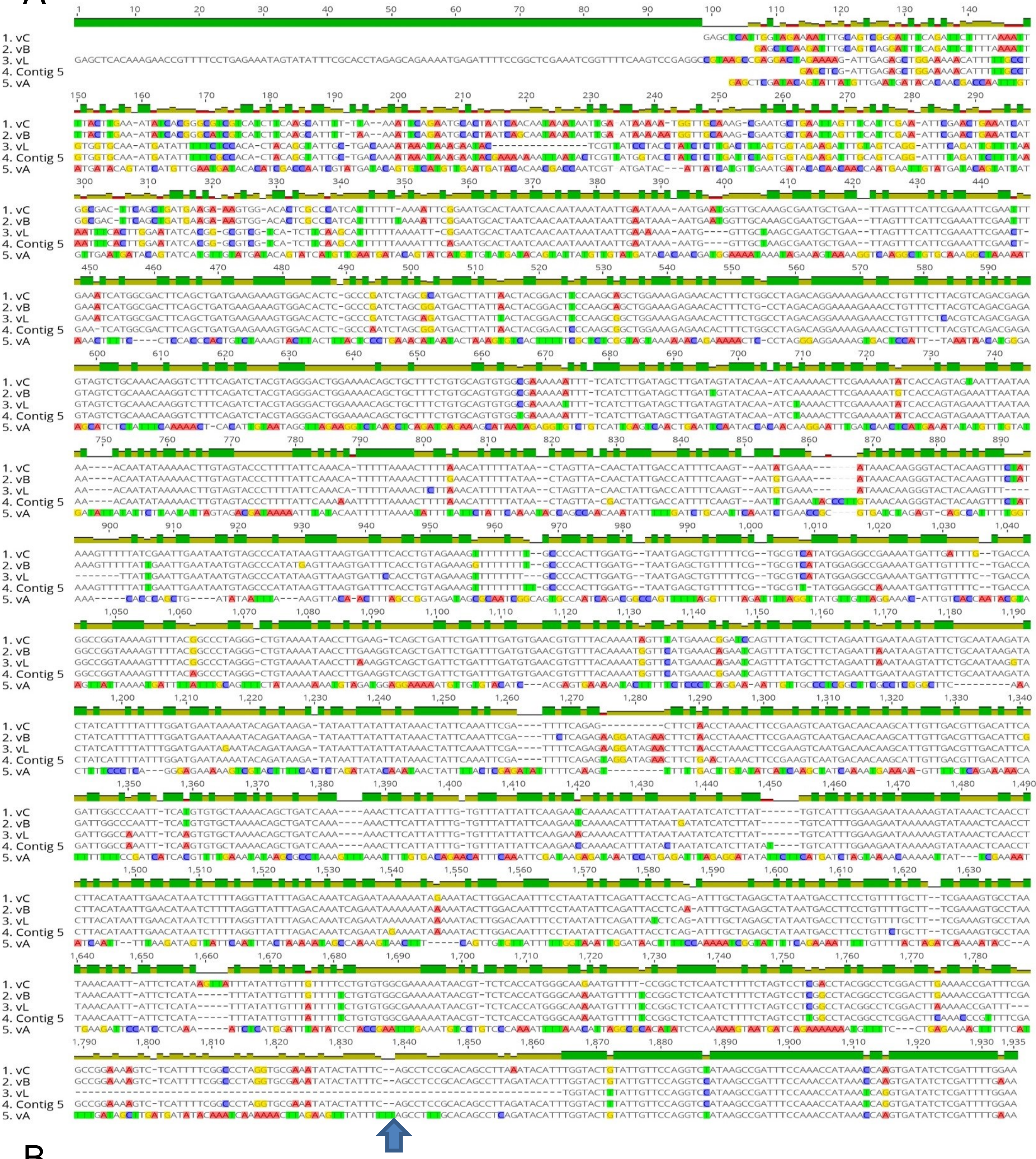

B

	vC	vB	vL	Contig 5	vA
vC		96.708%	82.986%	88.199%	45.985%
vB	96.708%		84.847%	89.128%	45.985%
vL	82.986%	84.847%		90.557%	43.022%
Contig 5	88.199%	89.128%	90.557%		46.340%
vA	45.985%	45.985%	43.022%	46.340%	

Figure S4. **Alignment of *CYP6ER1* promoter variants. Related to Figure 3.** A) Alignment of 1750bp of the putative promoter region upstream of different *CYP6ER1* variants. Contig 5 could not be assigned to a known *CYP6ER1* variant. The position of the sequence breakpoint upstream of *CYP6ER1vA* is illustrated with an arrow. B) Matrix of calculated sequence identity of the different promoter variants.

Name	Year collected	Country of origin	Region/area
**NLS**	1984	Japan	Unknown
**NLF1**	August 2009	Thailand	Unknown
**NLF2**	November 2010	Vietnam	Trà Vinh Province, Southern Vietnam
**NLF3**	August 2011	Vietnam	Hau Giang
**NLF4**	August 2011	Indonesia	Anjatan District, Indramayu
**NLF5**	September 2011	India	Raipur, Chhattisgarth
**NLF6**	March 2012	India	Koppal District, Karnataka State
**NLF7**	April 2012	India	East Godavari District, Andhra Pradesh
**NLF8**	September 2012	India	Sidhikerra, Karnataka State

Table S1. Origin of BPH strains used in this study. Related to Figure 1.

Strain	LC50-value [mg/L^{-1}]	95% limits	Slope (+/- SD)	RR	Mortality 4 mg L^{-1}	Mortality 20 mg L^{-1}
**NLS**	0.6	0.50-0.70	1.822+/- 0.158	1	-	-
**NLF1**	111.7	66.05-212	0.891+/- 0.11	186.2	-	-
**NLF2**	170.2	46.44-5095	0.655+/- 0.221	283.7	-	-
**NLF3**	>1000	-	-	1666	-	-
**NLF4**	-	-	-	-	16.50 (+/-8.42)	20.20 (+/-4.21)
**NLF5**	-	-	-	-	30.14 (+/-8.11)	34.25 (+/-8.14)
**NLF6**	-	-	-	-	15.13 (+/-5.67)	58.31 (+/-8.00)
**NLF7**	14.49	4.55-30.81	0.653+/- 0.134	24.2	-	-
**NLF8**	-	-	-	-	15.74 (+/-5.69)	42.42 (+/-8.02)

Table S2. Sensitivity of susceptible and resistant strains of *Nilaparvata lugens* to imidacloprid topically applied to adult females. **Related to Figure 1**.
Data for strain NLS, NLF1, 2, 3, and 7 shows lethal concentrations 50% (LC_{50} values) determined by probit analyses. Data for NL4, 5, 6, and 8 shows percentage mortality (± standard error) at two diagnostic doses (LD_{95} and 5XLD_{95} of the susceptible strain).

Oligo	Sequence	Purpose
ER1 CDS F1	ATGTGGGAAAACTCGTGGTTGG	PCR/sequencing of CYP6ER1 coding sequence
ER1 CDS F2	GGTTGGCCTAYCTTGTCACAGG	PCR/sequencing of CYP6ER1 coding sequence
ER1 CDS R1	CTAAGTATCTCTCGCTACCAGC	PCR/sequencing of CYP6ER1 coding sequence
ER1 CDS R2	GCTACCAGCTTCAGTGTGAGG	PCR/sequencing of CYP6ER1 coding sequence
CYP6ER1 vL/vF F	CATCCATGAGGTCTACGAAG	CYP6ER1 variant-specific QPCR
CYP6ER1 vL/vF R	GAGTGCTGAACAGATGGTGT	CYP6ER1 variant-specific QPCR
CYP6ER1 vA F	CTTTCTTCACCCCCGCCC	CYP6ER1 variant-specific QPCR
CYP6ER1 vA R	CCTGCATGGTCTCGAACATG	CYP6ER1 variant-specific QPCR
CYP6ER1 vB F	TCTTGTCACAATCCTGTTGCTG	CYP6ER1 variant-specific QPCR
CYP6ER1 vB R	TGGATGCATTTCTTGGACAATACG	CYP6ER1 variant-specific QPCR
CYP6ER1 vC F	GAGACTACTTCTGCATCTTTGT	CYP6ER1 variant-specific QPCR
CYP6ER1 vC R	GGAAACCATTGGGAAGAATGA	CYP6ER1 variant-specific QPCR
CYP6ER1 vD F	AGATCAAATCGGCGGATGGA	CYP6ER1 variant-specific QPCR
CYP6ER1 vD R	CGGAATCATCACTTGAGTTCC	CYP6ER1 variant-specific QPCR
CYP6ER1 vE R	CCGGAATCATTACTTGAGTTCC	CYP6ER1 variant-specific QPCR
CYP6ER1 vE F	GTATGATGAGATCAGATCTGTGA	CYP6ER1 variant-specific QPCR
D099 pUAST F	TCACTGGAACTAGGCTAGCA	Sequence validation of transgenic flies
D102 pUAST F	GGATCCAAGCTTGCATGCCTG	Sequence validation of transgenic flies
D100 pUAST R	AAAGGCATTCCACCACTGCT	Sequence validation of transgenic flies
D101 pUAST R	CCACCACTGCTCCCATTCAT	Sequence validation of transgenic flies
ER1 deletion F	GCAGAAATGTTGAGGAAATATCCC	Sequencing of exon 6 of BPH individuals
ER1 deletion R1	ACCAAAGGGTATGAAAGAGAAGG	Sequencing of exon 6 of BPH individuals
Ex3 qPCR F2	GAATGTGATTGCCTCCACGG	Copy number QPCR of CYP6ER1
Ex3 qPCR R2	AGCATCAGCAAGTGGGTTCT	Copy number QPCR of CYP6ER1
Ex4 qPCR F2	AACATGAGGTTCACGCCGAA	Copy number QPCR of CYP6ER1
Ex4 qPCR R2	TGCATGAAATCCTTCCTCACCA	Copy number QPCR of CYP6ER1
VGSC qPCR F2	CACCATTGTCACACAGCAGC	qPCR of reference gene (VGSC)
VGSC qPCR R2	CCCTGGAGTAGTGCTTGTCG	qPCR of reference gene (VGSC)
Nl_Actin_F	TAACGAGAGGTTCCGTTGCC	qPCR of reference gene (actin)
Nl_Actin_R	GACAGGACAGTGTTGGCGTA	qPCR of reference gene (actin)
Nl_α2_tubulin_F	CCACCCTGGAACACTCTGAC	qPCR of reference gene (α2_tubulin)
Nl_α2_tubulin_R	CGAAGCAGTGATCGAGGACA	qPCR of reference gene (α2_tubulin)

Table S3. Sequence of oligonucleotide primers used in this study. **Related to STAR methods**.
