# Supplementary material for: Neofunctionalization of Duplicated P450 Genes Drives the Evolution of Insecticide Resistance in the Brown Planthopper
Source: Curr Biol. 2018 Jan 22;28(2):268–274.e5. doi: 10.1016/j.cub.2017.11.060 (PMC5788746; doi:10.1016/j.cub.2017.11.060)
Supplement: Document S2. Article plus Supplemental Information [file mmc2.pdf]

Current Biology

Report

Neofunctionalization of Duplicated P450 Genes Drives the Evolution of Insecticide Resistance in the Brown Planthopper

Highlights

- The cytochrome P450 *CYP6ER1* is duplicated in imidacloprid resistant *N. lugens* strains
- Amino-acid alterations in certain CYP6ER1 variants confer resistance to imidacloprid
- Resistant hoppers have paralogs with and without the gain-of-function mutations
- The susceptible and mutant *CYP6ER1* copies show marked divergence in their expression

Authors

Christoph T. Zimmer, William T. Garrood, Kumar Saurabh Singh, ..., Ralf Nauen, T.G. Emyr Davies, Chris Bass

Correspondence

c.bass@exeter.ac.uk

In Brief

Zimmer et al. explore the functional significance of genetic variation at the loci encoding CYP6ER1, a cytochrome P450 enzyme, in field strains of the brown planthopper. They show that duplication of *CYP6ER1* provided opportunities for functional and regulatory innovation, leading to resistance to the insecticide imidiacloprid.

Zimmer et al., 2018, Current Biology *28*, 268–274
January 22, 2018 © 2017 The Authors. Published by Elsevier Ltd.
https://doi.org/10.1016/j.cub.2017.11.060

Current Biology
Report

Neofunctionalization of Duplicated P450 Genes Drives the Evolution of Insecticide Resistance in the Brown Planthopper

Christoph T. Zimmer,[1,4,5] William T. Garrood,[2,4] Kumar Saurabh Singh,[1] Emma Randall,[1] Bettina Lueke,[3] Oliver Gutbrod,[3] Svend Matthiesen,[3] Maxie Kohler,[3] Ralf Nauen,[3] T.G. Emyr Davies,[2] and Chris Bass[1,6,*]
[1]College of Life and Environmental Sciences, Biosciences, University of Exeter, Penryn Campus, Penryn, Cornwall TR10 9FE, UK
[2]Department of Biointeractions and Crop Protection, Rothamsted Research, Harpenden AL5 2JQ, UK
[3]Bayer AG, Crop Science Division, Alfred Nobel-Strasse 50, 40789 Monheim, Germany
[4]These authors contributed equally
[5]Present address: Syngenta Crop Protection, Werk Stein, Schaffhauserstrasse, Stein CH4332, Switzerland
[6]Lead Contact
*Correspondence: c.bass@exeter.ac.uk
https://doi.org/10.1016/j.cub.2017.11.060

SUMMARY

Gene duplication is a major source of genetic variation that has been shown to underpin the evolution of a wide range of adaptive traits [1, 2]. For example, duplication or amplification of genes encoding detoxification enzymes has been shown to play an important role in the evolution of insecticide resistance [3–5]. In this context, gene duplication performs an adaptive function as a result of its effects on gene dosage and not as a source of functional novelty [3, 6–8]. Here, we show that duplication and neofunctionalization of a cytochrome P450, CYP6ER1, led to the evolution of insecticide resistance in the brown planthopper. Considerable genetic variation was observed in the coding sequence of *CYP6ER1* in populations of brown planthopper collected from across Asia, but just two sequence variants are highly overexpressed in resistant strains and metabolize imidacloprid. Both variants are characterized by profound amino-acid alterations in substrate recognition sites, and the introduction of these mutations into a susceptible P450 sequence is sufficient to confer resistance. *CYP6ER1* is duplicated in resistant strains with individuals carrying paralogs with and without the gain-of-function mutations. Despite numerical parity in the genome, the susceptible and mutant copies exhibit marked asymmetry in their expression with the resistant paralogs overexpressed. In the primary resistance-conferring CYP6ER1 variant, this results from an extended region of novel sequence upstream of the gene that provides enhanced expression. Our findings illustrate the versatility of gene duplication in providing opportunities for functional and regulatory innovation during the evolution of an adaptive trait.

RESULTS

We previously demonstrated that resistance to the insecticide imidacloprid in the brown planthopper (BPH), *Nilaparvata lugens*, is associated with the overexpression of the cytochrome P450, CYP6ER1 [9]. To explore if qualitative changes in this P450 also play a role in resistance, we first sequenced the complete coding cDNA of *CYP6ER1* in field populations of BPH collected from across Asia that all exhibit resistance to imidacloprid (Tables S1 and S2). Comparison of the sequences obtained with a reference sequence (*CYP6ER1vL*) derived from a lab-susceptible strain (NLS, Table S1) identified a total of 114 polymorphic sites that result in 27 amino-acid alterations (Figures 1A and S1A). These nucleotide sequences resolved to seven unique amino acid sequence variants: CYP6ER1vA, CYP6ER1vB, CYP6ER1vC, CYP6ER1vD1, CYP6ER1vD2, CYP6ER1vE, and CYP6ER1vF (Figures 1A, S1A, and S1B).

The relative expression of the different *CYP6ER1* variants in field populations of BPH was assessed by cDNA cloning and sequencing and using variant-specific qPCR. This revealed that just two of the *CYP6ER1* variants are highly expressed in strains from field populations: *CYP6ER1vA* was the major variant expressed in strains from Thailand, Vietnam, and Indonesia, whereas *CYP6ER1vB* was expressed in strains originating from India (Figures 1B and 1C). The expression of one predominant sequence variant in imidacloprid resistant populations of BPH in these regions suggests that *CYP6ER1vA* and *CYP6ER1vB* may play a primary role in resistance.

To test this, we expressed CYP6ER1vA, CYP6ER1vB, the lab susceptible variant CYP6ER1vL, and its closest relatives observed in the field, CYP6ER1vF and CYP6ER1vC, *in vitro* and examined their capacity to metabolize imidacloprid. Liquid chromatography tandem mass spectrometry (LC-MS/MS) analysis demonstrated that CYP6ER1vA and, to a lesser extent, CYP6ER1vB are effective metabolizers of imidacloprid, converting it to 4/5-hydroxy imidacloprid and 6-chloronicotinic acid (6-CNA) (Figure 1D). In contrast, no significant metabolism of imidacloprid was observed in the case of CYP6ER1vC, CYP6ER1vL, or CYP6ER1vF (Figure 1D).

 © 2017 The Authors. Published by Elsevier Ltd.
This is an open access article under the CC BY license (http://creativecommons.org/licenses/by/4.0/).

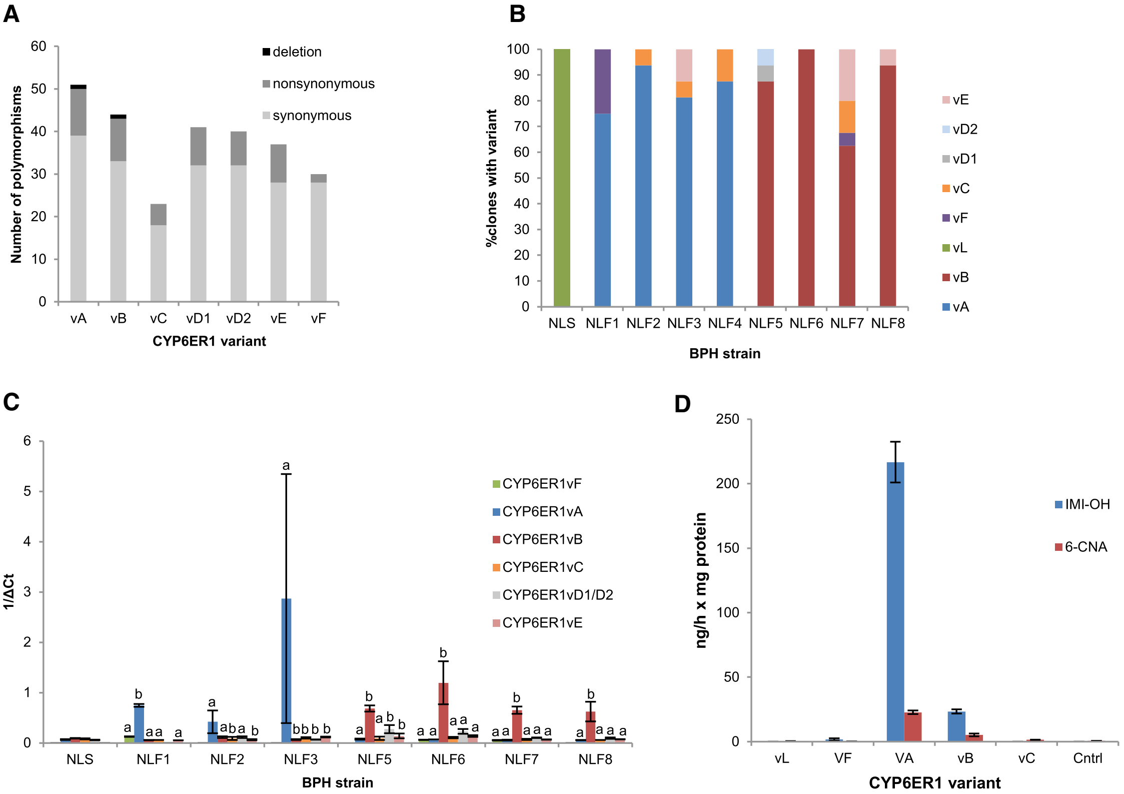

Figure 1. Characterization of *CYP6ER1* Variants in BPH Populations

(A) Number and type of nucleotide polymorphisms in different CYP6ER1 variants relative to CYP6ER1vL, the variant observed in the lab-susceptible strain.

(B and C) Relative expression of CYP6ER1 variants in imidacloprid-resistant BPH field strains (NLF1-8) and a susceptible strain (NLS), as determined by cDNA cloning and sequencing (B), and variant-specific QPCR (C). In (C), letters above bars are used to denote significant (p = < 0.01 in all cases) differences in expression between variants within each strain as assessed by one-way ANOVA with post-hoc Tukey HSD. Error bars in (C) indicate 95% confidence intervals (n = 4).

(D) Metabolism of imidacloprid by recombinantly expressed CYP6ER1 variants. NADPH-dependent conversion of imidacloprid to 4/5-hydroxy imidacloprid (IMI-OH) and 6-chloronicotinic acid (6-CNA) is shown. Error bars indicate 95% confidence intervals (n = 3).

See also Figure S1 and Tables S1 and S2.

To explore which amino acid polymorphisms in CYP6ER1vA and CYP6ER1vB are responsible for imidacloprid metabolism, we first mapped polymorphisms in all CYP6ER1 variants to important known P450 domains (Figures 2A and S1A). This highlighted two features unique to these variants. First, both CYP6ER1vA and CYP6ER1vB share an amino-acid substitution in substrate recognition site (SRS) 4 at position 318, where a threonine in all other variants is replaced with a serine. Significantly, this occurs at a highly conserved position in a P450 signature sequence [A/G]GX[E/D]T[T/S] in helix I, known as the oxygen-binding motif. The second alteration unique to CYP6ER1vA/B is the deletion of an amino acid in SRS5 in both variants. In CYP6ER1vA, this occurs at Ala375 and is immediately followed by an alanine to glycine substitution, whereas in CYP6ER1vB, a proline is deleted at position 377. To predict the effect of these amino acid changes on imidacloprid binding, CYP6ER1 was computationally modeled and docking simulations of imidacloprid within the active site of CYP6ER1vL, CYP6ER1vA, and CYP6ER1vB performed (Figures 2B, 2C, and 2D). This revealed that T318 and A376, along with additional residues, create the hydrophobic interface of the binding cavity (Figure 2B). In CYP6ER1vA, T318S in combination with A376G increases the conformational space accessible to imidacloprid between these two positions (Figure 2C). Although less pronounced, this is also true for CYP6ER1vB, where the T318S mutation comes together with an alanine at position 376 (Figure 2D). In this instance, due to the proline deletion, the fold of CYP6ER1vB is shifted slightly away from the I-helix substitution with the consequence of a similar gap opening. These alterations are consistent with the hydroxylation capacity seen for individual CYP6ER1 variants when functionally expressed and are strong candidates for the gain-of-function observed.

To validate the predictions made by homology modeling, we employed site-directed mutagenesis in combination with functional expression *in vivo*. For this, a series of amino-acid alterations were introduced into the susceptible CYP6ER1vL sequence as follows: T318S, P377del, A375del+A376G, T318S+P377del, and T318S+A375del+A376G. A series of transgenic *Drosophila* lines were created that ubiquitously express each of these mutated P450s, or the wild-type variant CYP6ER1vL, and their sensitivity to imidacloprid was assessed (Table 1). The T318S substitution, shared by both variants, resulted in a marked (20-fold) and significant increase in resistance

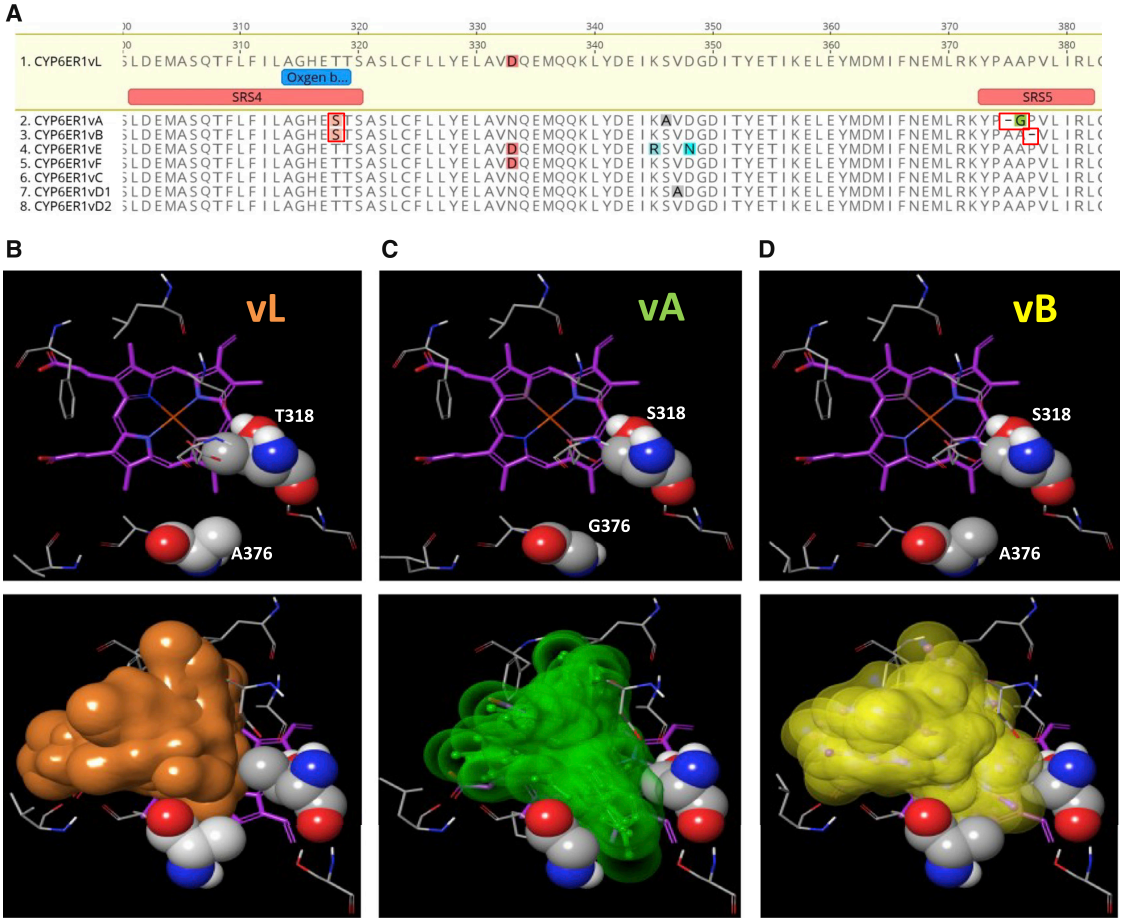

Figure 2. Modeling the Active Site of CYP6ER1 Reveals the Impact of Amino Acid Alterations on Imidacloprid Binding

(A) Amino-acid alignment of CYP6ER1 variants highlighting substitutions and deletions within substrate recognition sites four and five (boxed in red).

(B–D) Protein homology modeling for 3 different CYP6ER1 variants (upper row), showing key residues surrounding the catalytic site. Amino-acid positions T318/A376 in CYP6ER1vL (B), S318/G376 in CYP6ER1vA (C), and S318/A376 in CYP6ER1vB (D) are in close proximity (spacefill representation). Imidacloprid docking into the active site is illustrated (lower row) by colored volumes, constituting an envelope around an ensemble of possible binding poses.

See also Figure S1.

compared to the wild-type susceptible variant. Deletion of Pro377, as seen in CYP6ER1vB, provided a more moderate, but significant, 4.5-fold increase in resistance. In contrast, the A375del+A376G alteration observed in CYP6ER1vA conferred higher levels (20-fold) of resistance to imidacloprid. When T318S was combined with P377del (as seen in CYP6ER1vB), an epistatic interaction was observed, with the resistance conferred by the double mutation (20-fold) less than the sums of the effects of the component single mutations. In contrast, an additive interaction was observed when T318S was combined with A375del+A376P (as observed in CYP6ER1vA), with this combination exhibiting the highest resistance of all mutant lines (35-fold). These data are consistent with the relative efficiency of imidacloprid hydroxylation by CYP6ER1vA and CYP6ER1vB observed *in vitro* (Figure 2D) and convincingly demonstrate the adaptive nature of the genetic alterations observed in these isozymes.

To examine if the high levels of genetic variation in *CYP6ER1* in BPH field populations results, in part, from gene copy number variation (CNV), we resequenced the genomes of three BPH strains that primarily express *CYP6ER1vA* (NLF2), *CYP6ER1vB* (NLF7), or *CYP6ER1vL* (NLS), respectively. Reads were mapped to the coding sequence of *CYP6ER1* and two single-copy reference genes in the genome of BPH: the P450 *CYP6AY1* and the voltage-gated sodium channel (*VGSC*); and read coverage across each gene was compared between strains (Figures 3A–3F). No significant shift in coverage was observed between the three strains across *CYP6AY1* or *VGSC*. However, an approximately 2-fold increase in read depth was observed over the coding sequence of *CYP6ER1* between the two resistant strains NLF2 and NLF7 and the susceptible strain NLS, suggesting that *CYP6ER1* is duplicated in the field strains. qPCR confirmed this finding, with the copy number of *CYP6ER1* 2.2-fold higher in NLF7 and 1.9-fold higher in NLF2 than in NLS. The mean cycle threshold values in qPCR of *CYP6ER1* and the single-copy reference gene in NLS were essentially the same (23.50 [SEM = 0.04] and 23.62 [SEM = 0.08], respectively), indicating that *CYP6ER1* is present as a single copy in the haploid genome of NLS and two copies in NLF2 and NLF7 (Figures 3G and 3H).

Table 1. Log-Dose Probit-Mortality Data for Imidacloprid against Female Transgenic Drosophila-Expressing CYP6ER1 Mutants

Strain	LC50 [mg/L^{-1}]	95% CL	Slope (+/− SD)	Resistance Ratio to vL
No transgene	111.1	45-233	1.373 ± 0.287	-
Wildtype (vL)	53.1	31.4-82.6	1.864 ± 0.328	-
T318S	1062	531-2292	1.103 ± 0.183	20.0
P377del	237	101.4-520	1.324 ± 0.262	4.5
A375del+A376G	1062	752-1501	2.082 ± 0.264	20.0
T318S+P377del	1063	442-2680	1.54 ± 0.356	20.0
T318S+A375del+A376G	1857	905-4229	1.448 ± 0.293	35.0

The wildtype reference line expresses CYP6ER1vL. No transgene: Flies of the same genetic background but minus the transgene.

To explore if one or both of the gene copies in resistant BPH individuals carry the resistance-conferring mutations, ~30 individuals of the NLF2 and NLF7 strains were genotyped by PCR and direct sequencing. All individuals of the two strains scored as heterozygous for the diagnostic resistance mutations that characterize *CYP6ER1vA* and *CYP6ER1vB*, respectively (Figures S2A and S2B). This observed excess of apparent heterozygosity is a characteristic signature of a duplicated gene and demonstrates that individuals of both strains carry *CYP6ER1* copies with and without the resistance conferring mutations. To more precisely confirm which variants are present in these two strains, we cloned and sequenced amplified products from three individuals of each strain. For individuals of NLF2 we recovered two different sequences in equal abundance; the first carried the indels/SNPs that define *CYP6ER1vA*, while the second carried SNPs diagnostic for *CYP6ER1vC* (Figures 3I, S2C, and S2D). Sequencing of colonies from NLF7 individuals again recovered two alternative sequences in equal abundance: the first corresponded to *CYP6ER1vB*, while the SNP profile of the second most closely matched *CYP6ER1vF* (Figures 3I, S2C, and S2D). Thus, taken together with functional analyses (Figure 1D), these data reveal that the genomes of individuals of both resistant strains have *CYP6ER1* copies that encode the capacity to metabolise imidacloprid and those that do not.

To examine the expression of the different gene copies observed in NLF2 and NLF7, we repeated these experiments but used RNA extracted from individuals of each strain as template in RT-PCR. Again, we only observed sequences representing *CYP6ER1vA* and *CYP6ER1vC* in individuals of NLF2; however, *CYP6ER1vA* represented > 90% of the clones sequenced (Figures 3I and S2D). Similarly, in the case of NLF7, we only observed sequences representing *CYP6ER1vB* and *CYP6ER1vF*, with the former representing > 90% of the sequences recovered (Figures 3I and S2D). Thus, the duplicate genes exhibit marked divergence in their expression, with the copy with the gain-of-function mutations overexpressed.

To explore the molecular basis of this and simultaneously examine the genomic architecture of different CYP6ER1 variants, we employed a gene capture approach. This allowed the close-to-complete gene sequence of *CYP6ER1vL*, *CYP6ER1vA*, *CYP6ER1vB*, *CYP6ER1vC*, and *CYP6ER1vF* to be assembled (Figure 3J). Considerable variation was seen in intron size and sequence between different CYP6ER1 variants (Figures 3J and S3). This was particularly pronounced in the case of *CYP6ER1vA* and *CYP6ER1vB*. For example, in *CYP6ER1vA*, intron 1 is > 4.9 kb in size in contrast to ~4 kb in other variants, and intron 4 is just 316 bp in size compared to an average size of ~5 kb in other variants. In the case of *CYP6ER1vB*, intron 2 was much larger than in all other variants (5,478 bp compared to an average of 3,302 bp), with this expanded intron size resulting from an internal duplication that included the last 123 bp of exon 3. This pseudo-exon was never observed in *CYP6ER1vB* transcripts, likely due to the loss of the splice site consensus sequence upstream of the duplicated region.

Five putative promoter variants were identified by gene capture, four of which could be linked to *CYP6ER1vA*, *CYP6ER1vB*, *CYP6ER1vC*, and *CYP6ER1vL* (Figures 3K and S4). Surprisingly, only the promoter associated with *CYP6ER1vA* matched the sequence upstream of *CYP6ER1* on scaffold KN153994.1 of the reference genome [10]. The remaining four promoter variants do not show sequence similarity with any other scaffold in the genome. Indeed, alignment of the promoter variants reveals a clear break point 104 bp upstream of the start codon in *CYP6ER1vA*, with the sequence of this variant completely diverging from the other four variants after crossing this point (Figures 3K and S4). We used reporter gene assays to explore the effect of this on gene expression. No significant differences were seen in reporter gene expression driven by a ~1.8 kb region of the promoters of *CYP6ER1vB*, *CYP6ER1vC*, *and CYP6ER1vL* (Figure 3L). This finding suggests that the overexpression of *CYP6ER1vB* seen in resistant BPH strains results from either *trans*-acting factors or *cis*-acting elements outside of the region analyzed. In contrast, we observed a significant (up to 9.5-fold) increase in expression driven by the promoter of *CYP6ER1vA* in comparison to all other promoter variants (Figure 3L). This suggests that *cis*-acting elements in the region upstream of *CYP6ER1vA*, derived from the novel genomic sequence, are responsible for the high expression of this variant in BPH populations across Southeast Asia.

DISCUSSION

Our data show that two CYP6ER1 sequence variants are highly expressed in imidacloprid resistant field populations of BPH from across Asia. Both variants are defined by the same or similar mutations that confer the capacity to metabolise imidacloprid but, despite this, appear to have independent origins—CYP6ER1vA evolving in southeast Asia and CYP6ER1vB in India. Cases of parallel evolution can shed light on the repeatability of evolution while also providing insight into molecular constraints. In the case of CYP6ER1, the repeated acquisition of amino-acid alterations at the same or similar sites suggests

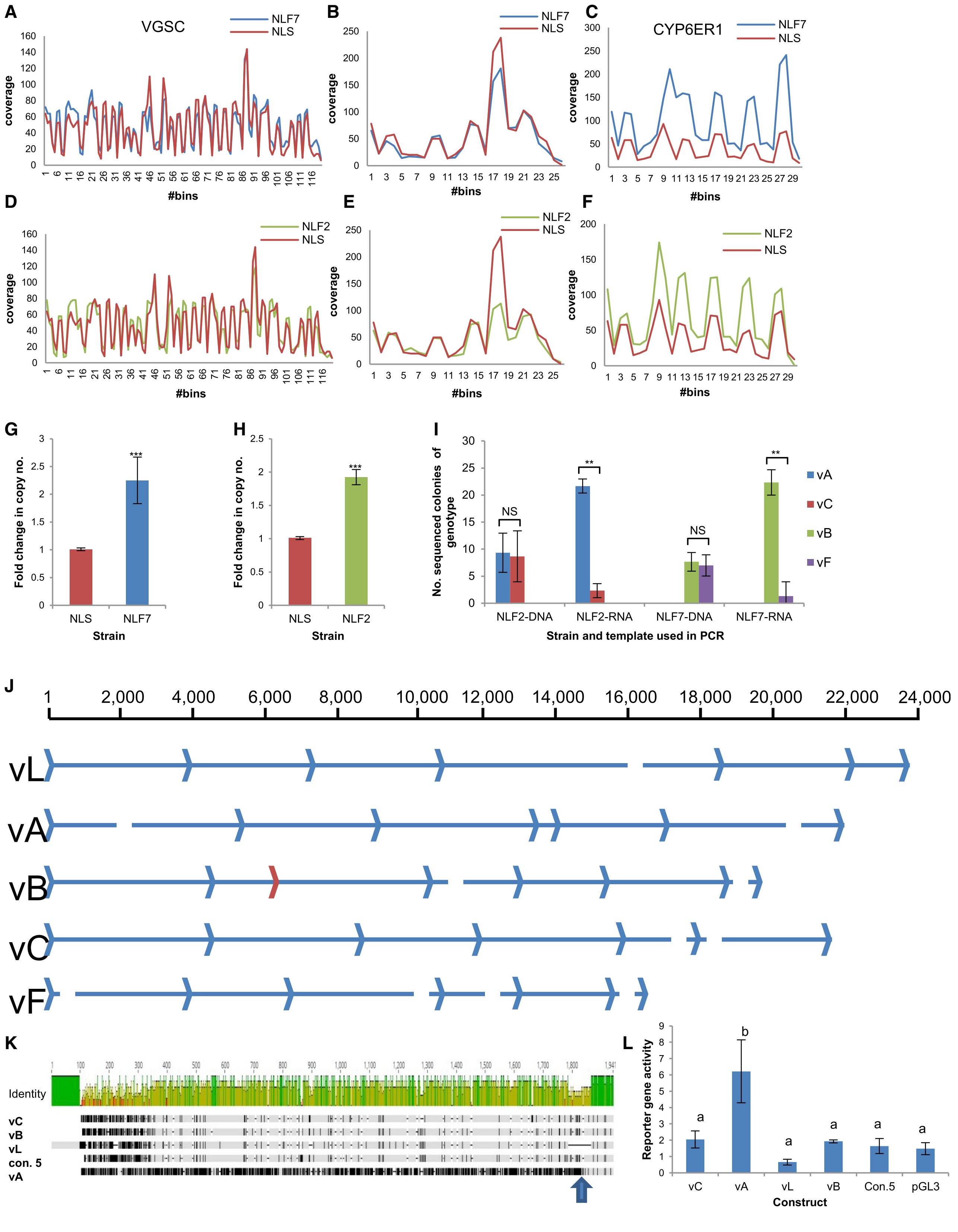

(legend on next page)

that only modification of these sites bring about the functional change in imidacloprid sensitivity while satisfying other constraints.

We demonstrate that *CYP6ER1* is duplicated in resistant strains and show that resistant individuals carry one copy with the gain-of-function mutations and one without. In most examples of gene duplication or amplification of detoxification, enzymes associated with metabolic resistance duplicates are identical and are retained because of the clear benefits of increased gene dosage [3, 4, 7, 8]. In contrast, in this example, copying of the ancestral *CYP6ER1* gene would not immediately provide a fitness benefit in the presence of insecticide, as it lacks the capacity to metabolise imidacloprid. Rather, the evolution of the novel, selectively beneficial function of the mutant copy of *CYP6ER1*, which was not present in the ancestral gene, is a compelling example of neofunctionalization [1]. In the classical model outlined by Ohno, gene duplication creates redundancy, allowing the second gene copy, under relaxed constraint, to accrue mutations, which, if adaptive, are fixed under selection [11]. Several of our findings are consistent with this model and suggest that gene duplication was required to free CYP6ER1 from functional constraint and permit the acquisition of mutations that led to resistance. First, resistant BPH individuals retain a wild-type copy of CYP6ER1 despite the fact that it encodes an enzyme with no capacity to metabolise imidacloprid, suggesting that it is important for organismal fitness. Second, the genetic alterations seen in *CYP6ER1vA* and *CYP6ER1vB* are profound, resulting in the substitution of a highly conserved residue in SRS4 and the deletion of amino acids in SRS5. Although the native substrate(s) of CYP6ER1 is unknown, it is reasonable to predict that the nature and location of these alterations would alter the binding and subsequent enzymatic conversion of the natural substrates of this enzyme. Finally, comparison of *CYP6ER1* from BPH and its orthologs in white-backed planthopper (*Sogatella furcifera*) and small brown planthopper (*Laodelphax striatellus*) reveal that, while the two orthologous P450s diverge from *CYP6ER1* at > 30% of amino acids, they are completely conserved at the site of the resistance mutations, where they all have the wild-type residues, suggesting that these sites are highly constrained.

Gene capture analyses revealed marked changes in the genomic architecture of *CYP6ER1vA* and *CYP6ER1vB* compared to other variants, including the putative single-copy ancestor *CYP6ER1vL*, strongly suggesting that the duplication of *CYP6ER1* predates the introduction of imidacloprid in the early 1990s. Thus, gene duplication itself was not a *de novo* mutation occurring in response to insecticide use. Rather, our data are most parsimonious with a model of evolutionary opportunism with the exaptation of existing standing genetic variation (in this case, CNV), when the environmental conditions changed, facilitating subsequent functional innovation.

A central question in evolutionary biology is the relative contribution of functional versus regulatory divergence during the evolution of new genes. In this regard, a significant finding of our study was that, although resistant BPH individuals carry copies of *CYP6ER1* with and without resistance mutations, only the mutant copy is highly expressed. We provide a molecular explanation for this in the case of *CYP6ER1vA* with gene capture revealing an extended region of novel sequence upstream of this variant in comparison to all other variants. The extent and size of this region of divergent sequence is consistent with a duplication breakpoint, although we cannot exclude a large indel upstream of this variant as an alternative possibility. Reporter genes assays demonstrated that the novel sequence provides *cis*-acting elements that result in the increased expression of *CYP6ER1vA*. In the presence of insecticide, this increased expression would be highly beneficial, likely explaining, in part, why *CYP6ER1vA* is now the predominant variant expressed in resistant BPH populations in Asia.

In summary, we provide a novel example of the evolution of metabolic resistance by gene duplication and neofunctionalization. In this case study, the chromosomal rearrangements involved provided opportunities for functional and regulatory innovation, once again highlighting the remarkable capacity of gene duplication to drive the evolution of adaptive traits.

STAR★METHODS

Detailed methods are provided in the online version of this paper and include the following:

- KEY RESOURCES TABLE
- CONTACT FOR REAGENT AND RESOURCE SHARING
- EXPERIMENTAL MODEL AND SUBJECT DETAILS
    - Insect strains
    - Insect cell lines

Figure 3. Genomic Analyses of the *CYP6ER1* Locus

(A–F) Coverage plots of DNA-seq reads from the NLS (imidacloprid susceptible) strain and NLF7 and NLF2 (imidacloprid resistant) strains mapped to the coding sequence of two reference single-copy genes: the voltage-gated sodium channel (VGSC) (A and D) and *CYP6AY1* (B and E), and *CYP6ER1* (C and F).

(G and H) Copy number of *CYP6ER1* in the NLF7 (G) and NLF2 (H) strains relative to NLS determined by qPCR. Error bars indicate 95% confidence intervals (n = 4). ***$p < 0.001$; one-way ANOVA with post hoc Tukey HSD.

(I) Number of sequenced colonies obtained of each *CYP6ER1* variant after cloning and sequencing PCR products amplified from either genomic DNA or mRNA of individuals of the NLF2 and NLF7 strains. Error bars indicate 95% confidence limits (n = 3). NS, not significant. **$p < 0.01$; paired t test.

(J) Assembly of gene capture long reads reveals marked variation in intron size between different variants. The position of exons is shown by blue arrow heads, with the partially duplicated exon in *CYP6ER1vB* highlighted in red. Gaps illustrate the position of assembly gaps.

(K) Alignment of different putative promoter variants of *CYP6ER1* upstream of the translation start codon. Gray regions indicate similarity between sequences, and black regions indicate sequence differences. Indels are indicated by gaps in the sequences. The identity plot above the alignment displays the identity across all sequences for every position. Green indicates that the residue at the position is the same across all sequences. Yellow is for less than complete identity, and red refers to very low identity for the given position. The position of the breakpoint observed in *CYP6ER1vA* is illustrated with an arrow.

(L) Reporter gene activity (normalized to renilla fluorescence) of *CYP6ER1* promoter variants. Letters above bars indicate significant differences, $p < 0.001$; one-way ANOVA with post-hoc Tukey HSD. Error bars indicate 95% confidence limits (n = 3).

See also Figures S2, S3, and S4.

- METHOD DETAILS
  - Identification and expression analysis of CYP6ER1 sequence variants
  - Heterologous expression of P450s
  - Metabolism assays and LC-MS/MS analysis
  - Homology modeling of CYP6ER1 and molecular docking simulation
  - Transgenic expression of candidate genes in *D. melanogaster*
  - Copy number analysis and sequencing of individual hoppers
  - Gene capture
  - Reporter gene assays
- QUANTIFICATION AND STATISTICAL ANALYSIS
- DATA AND SOFTWARE AVAILABILITY

SUPPLEMENTAL INFORMATION

Supplemental Information includes four figures and three tables and can be found with this article online at https://doi.org/10.1016/j.cub.2017.11.060.

ACKNOWLEDGMENTS

This project has received funding from the European Research Council (ERC) under the European Union's Horizon 2020 research and innovation programme (grant agreement n°646625), the Biotechnology and Biological Sciences Research Council of the UK (BB/G023352/1), and Bayer Crop Science.

AUTHOR CONTRIBUTIONS

Conceptualization, C.T.Z., and C.B.; Investigation, C.T.Z., W.T.Z., K.S.S., E.R., B.T., O.G., S.V.M., M.K., R.N., C.B.; Writing – Original Draft, C.B.; Writing – Review & Editing, all authors; Visualization: C.T.Z., K.S.S., R.N., and C.B.; Supervision, C.T.Z., R.N., T.G.E.D., and C.B.

Received: November 4, 2017
Revised: November 22, 2017
Accepted: November 23, 2017
Published: January 11, 2018

REFERENCES

1. Conant, G.C., and Wolfe, K.H. (2008). Turning a hobby into a job: how duplicated genes find new functions. Nat. Rev. Genet. *9*, 938–950.
2. Kondrashov, F.A. (2012). Gene duplication as a mechanism of genomic adaptation to a changing environment. Proc. Biol. Sci. *279*, 5048–5057.
3. Bass, C., Zimmer, C.T., Riveron, J.M., Wilding, C.S., Wondji, C.S., Kaussmann, M., Field, L.M., Williamson, M.S., and Nauen, R. (2013). Gene amplification and microsatellite polymorphism underlie a recent insect host shift. Proc. Natl. Acad. Sci. USA *110*, 19460–19465.
4. Field, L.M., and Devonshire, A.L. (1997). Structure and organization of amplicons containing the E4 esterase genes responsible for insecticide resistance in the aphid *Myzus persicae* (Sulzer). Biochem. J. *322*, 867–871.
5. Mouches, C., Pauplin, Y., Agarwal, M., Lemieux, L., Herzog, M., Abadon, M., Beyssat-Arnaouty, V., Hyrien, O., de Saint Vincent, B.R., Georghiou, G.P., et al. (1990). Characterization of amplification core and esterase B1 gene responsible for insecticide resistance in *Culex*. Proc. Natl. Acad. Sci. USA *87*, 2574–2578.
6. Field, L.M., Blackman, R.L., Tyler-Smith, C., and Devonshire, A.L. (1999). Relationship between amount of esterase and gene copy number in insecticide-resistant *Myzus persicae* (Sulzer). Biochem. J. *339*, 737–742.
7. Puinean, A.M., Foster, S.P., Oliphant, L., Denholm, I., Field, L.M., Millar, N.S., Williamson, M.S., and Bass, C. (2010). Amplification of a cytochrome P450 gene is associated with resistance to neonicotinoid insecticides in the aphid *Myzus persicae*. PLoS Genet. *6*, e1000999.
8. Schmidt, J.M., Good, R.T., Appleton, B., Sherrard, J., Raymant, G.C., Bogwitz, M.R., Martin, J., Daborn, P.J., Goddard, M.E., Batterham, P., and Robin, C. (2010). Copy number variation and transposable elements feature in recent, ongoing adaptation at the *Cyp6g1* locus. PLoS Genet. *6*, e1000998.
9. Bass, C., Carvalho, R.A., Oliphant, L., Puinean, A.M., Field, L.M., Nauen, R., Williamson, M.S., Moores, G., and Gorman, K. (2011). Overexpression of a cytochrome P450 monooxygenase, CYP6ER1, is associated with resistance to imidacloprid in the brown planthopper, *Nilaparvata lugens*. Insect Mol. Biol. *20*, 763–773.
10. Xue, J., Zhou, X., Zhang, C.X., Yu, L.L., Fan, H.W., Wang, Z., Xu, H.J., Xi, Y., Zhu, Z.R., Zhou, W.W., et al. (2014). Genomes of the rice pest brown planthopper and its endosymbionts reveal complex complementary contributions for host adaptation. Genome Biol. *15*, 521.
11. Ohno, S. (1970). Evolution by gene duplication (New York: Springer).
12. Garrood, W.T., Zimmer, C.T., Gorman, K.J., Nauen, R., Bass, C., and Davies, T.G.E. (2016). Field-evolved resistance to imidacloprid and ethiprole in populations of brown planthopper *Nilaparvata lugens* collected from across South and East Asia. Pest Manag. Sci. *72*, 140–149.
13. Tamura, K., Stecher, G., Peterson, D., Filipski, A., and Kumar, S. (2013). MEGA6: Molecular Evolutionary Genetics Analysis version 6.0. Mol. Biol. Evol. *30*, 2725–2729.
14. Livak, K.J., and Schmittgen, T.D. (2001). Analysis of relative gene expression data using real-time quantitative PCR and the 2(-Δ Δ C(T)) Method. Methods *25*, 402–408.
15. Phillips, I.R., and Shephard, E.A. (2006). Cytochrome P450 Protocols (Humana Press).
16. Schrodinger Release 2017-2. Schrödinger LLC, New York.
17. Biosolve, I.T. LeadIT http://www.biosolveit.de/LeadIT.
18. Hindle, S.A., Rarey, M., Buning, C., and Lengaue, T. (2002). Flexible docking under pharmacophore type constraints. J. Comput. Aided Mol. Des. *16*, 129–149.
19. Andrews, S. (2010). FastQC: a quality control tool for high throughput sequence data. http://www.bioinformatics.babraham.ac.uk/projects/fastqc.
20. Krueger, F. (2012) Trim Galore: A wrapper tool around Cutadapt and FastQC to consistently apply quality and adapter trimming to FastQ files, with some extra functionality for MspI-digested RRBS-type (Reduced Representation Bisufite-Seq) libraries. http://www.bioinformatics.babraham.ac.uk/projects/trim_galore/.
21. Li, H. (2013). Aligning sequence reads, clone sequences and assembly contigs with BWA-MEM. arXiv, arXiv:1303.3997v1 [q-bio.GN].
22. Xie, C., and Tammi, M.T. (2009). CNV-seq, a new method to detect copy number variation using high-throughput sequencing. BMC Bioinformatics *10*, 80.
23. Li, H., Handsaker, B., Wysoker, A., Fennell, T., Ruan, J., Homer, N., Marth, G., Abecasis, G., and Durbin, R.; 1000 Genome Project Data Processing Subgroup (2009). The Sequence Alignment/Map format and SAMtools. Bioinformatics *25*, 2078–2079.
24. Pfaffl, M.W. (2001). A new mathematical model for relative quantification in real-time RT-PCR. Nucleic Acids Res. *29*, e45.
25. Hackl, T., Hedrich, R., Schultz, J., and Förster, F. (2014). proovread: large-scale high-accuracy PacBio correction through iterative short read consensus. Bioinformatics *30*, 3004–3011.

STAR★METHODS

KEY RESOURCES TABLE

REAGENT or RESOURCE	SOURCE	IDENTIFIER
Chemicals, Peptides, and Recombinant Proteins		
NutriFly premix food:	SLS	Cat# FLY1034
Phusion HF DNA polymerase	Thermo Fisher	Cat# 10024537
SYBR Green JumpStart Taq Readymix	Sigma-Aldrich	Cat# S4438-500RXN
Bradford reagent	Sigma-Aldrich	Cat# B6916-500ML
Insect GeneJuice Transfection Reagent	Novagen	Cat# 71259-3
Critical Commercial Assays		
ISOLATE II RNA Mini Kit	Bioline	Cat# BIO-52073
SuperScript III Reverse Transcriptase kit	Invitrogen	Cat# 18080044
Strataclone Blunt PCR Cloning Kit	Agilent	Cat# 240207
Bac-to-Bac Baculovirus Expression System	GIBCO	Cat# 10359016
NADPH Regeneration system	Promega	Cat# V9510
Plant DNeasy Mini Kit	QIAGEN	Cat# 69104
Dual Luciferase Reporter assay system	Promega	Cat# E1910
Library Efficiency DHα Competent cells	Invitrogen	Cat# 18263012
GeneJet Plasmid Miniprep kit	Thermo Scientific	Cat# K0502
Deposited Data		
Genomic sequence	https://www.ncbi.nlm.nih.gov/genbank/	GenBank: MF970458
Genomic sequence	https://www.ncbi.nlm.nih.gov/genbank/	GenBank: MF970459
Genomic sequence	https://www.ncbi.nlm.nih.gov/genbank/	GenBank: MF970460
Genomic sequence	https://www.ncbi.nlm.nih.gov/genbank/	GenBank: MF970461
Genomic sequence	https://www.ncbi.nlm.nih.gov/genbank/	GenBank: MF970462
Genomic sequence	https://www.ncbi.nlm.nih.gov/genbank/	GenBank: MF970463
Experimental Models: Cell Lines		
Sf9	GIBCO	Cat# 11496015
High Five	GIBCO	Cat# B85502
Experimental Models: Organisms/Strains		
Nilaparvata lugens: NLS strain	Japan	This paper
Nilaparvata lugens: NLF1 strain	Thailand	This paper
Nilaparvata lugens: NLF2 strain	Trà Vinh Province, Southern Vietnam	This paper
Nilaparvata lugens: NLF3 strain	Hau Giang, Vietnam	This paper
Nilaparvata lugens: NLF4 strain	Anjatan District, Indramayu, Indonesia	This paper
Nilaparvata lugens: NLF5 strain	Raipur, Chhattisgarh, India	This paper
Nilaparvata lugens: NLF6 strain	Koppal District, Karnataka State, India	This paper
Nilaparvata lugens: NLF7 strain	East Godavari District, Andhra Pradesh, India	This paper
Nilaparvata lugens: NLF8 strain	Sidhikerra, Karnataka State, India	This paper

(*Continued on next page*)

Continued		
REAGENT or RESOURCE	SOURCE	IDENTIFIER
Drosophila melanogaster, genotype: 13-20 ["y^1w^{67c23}; P attP40 25C6," "1;2"]	University of Cambridge	Stock 13-20
Drosophila melanogaster, genotype: Act5C-GAL4 strain "y[1] w[*]; P(Act5C-GAL4-w)E1/CyO,""1;2"]	Bloomington Stock Centre	Act5C-GAL4
Oligonucleotides		
See Supplemental Materials	N/A	See Table S3
Recombinant DNA		
Cytochrome P450 variants	GeneArt, CA, USA	N/A
Cytochrome P450 reductase (CPR)	GeneArt, CA, USA	GenBank: Q07994
Gateway pDEST8 Vector	Invitrogen	Cat#11804010
pUASTattB40 Vector	Bischof et al., 2007	Gift from Jacob Riveron, Liverpool School of Tropical Medicine
pGL3-basic luciferase reporter vector	Promega	Cat# E1751
pRL-CMV *Renilla* luciferase control reporter vector	Promega	Cat# E2261
Software and Algorithms		
Geneious v 9.1.8	Biomatters	https://www.geneious.com/download/
MEGA v 6.0	[13]	www.megasoftware.net
Schrödinger Software Suite	Schrödinger	https://www.schrodinger.com/downloads/releases
LeadIT v 2.2.0	BioSolveIT	https://www.biosolveit.de/LeadIT/
Genstat v 16	VSN International	https://www.vsni.co.uk/software/genstat/
FastQC v 0.11.5	[19]	https://www.bioinformatics.babraham.ac.uk/projects/download.html#fastqc
Trim Galore! v 0.4.2	Babraham Institute	https://www.bioinformatics.babraham.ac.uk/projects/download.html#trim_galore
BWA-MEM (Burrows-Wheeler Aligner)	[21]	https://sourceforge.net/projects/bio-bwa/files/
R v 3.0	R Core Team, 2013	https://cran.r-project.org/bin/windows/base/old/
CNV-seq	[22]	http://tiger.dbs.nus.edu.sg/cnv-seq/
SAM tools	[23]	https://sourceforge.net/projects/samtools/files/
Proovread	[25]	https://github.com/BioInf-Wuerzburg/proovread
GraphPad Prism v 7	GraphPad Software	https://www.graphpad.com/
Other		
Nilaparvata lugens reference genome	[10]	GenBank: GCA_000757685.1

CONTACT FOR REAGENT AND RESOURCE SHARING

Further information and requests may be directed to and will be fulfilled by the Lead Contact, Chris Bass (chris.bass@exeter.ac.uk).

EXPERIMENTAL MODEL AND SUBJECT DETAILS

Insect strains

A laboratory-maintained strain of *N. lugens* exhibiting susceptibility to imidacloprid (NLS) and eight field strains of BPH collected from Thailand (NLF1), Vietnam (NLF2, NLF3), Indonesia (NLF4) and India (NLF5, NLF6, NLF7, NLF8) were reared in the laboratory on whole rice plants (*Oryza sativa* L. ssp.) under controlled environmental conditions (26°C/16h photoperiod). Year of collection is detailed in Table S1. We have previously shown that all eight of the field strains exhibit resistance to imidacloprid (Table S2) [12].

The *Drosophila melanogaster* stock 13-20 ["y^1w^{67c23}; P attP40 25C6," "1;2"] obtained from the University of Cambridge was used to create all transgenic lines. Virgin females of this line were crossed to males of the Act5C-GAL4 strain ["y[1] w[*]; P(Act5C-GAL4-w)E1/CyO,""1;2"] (Bloomington Stock Center) to activate transgene expression (see below for description of methods). All flies were reared on NutriFly food (NLS) at 24°C. Only female flies 2-5 days post eclosion were used for insecticide bioassays.

Insect cell lines

The Sf9 and High Five insect cell lines (ovarian cells from *Spodoptera frugiperda* and *Trichoplusia ni* respectively) were maintained in suspension culture under serum-free conditions at 27°C containing 25 μg/mL^{-1} gentamycin in SF-900 II SFM (GIBCO) and Express Five SFM (GIBCO), respectively.

METHOD DETAILS

Identification and expression analysis of CYP6ER1 sequence variants

Total RNA was extracted from 4 pools of 8 adult hoppers of each strain using the ISOLATE II RNA Mini Kit (Bioline) and reverse transcribed to cDNA using Superscript III reverse transcriptase (Invitrogen) following manufacturer protocols in both cases. Phusion DNA polymerase (Thermo) was used to amplify the full coding sequence of CYP6ER1 following the manufacturers protocol and using 10 ng of cDNA as template in 50 μl reactions and the primers listed in Table S4. Thermocycling conditions consisted of an initial denaturation step at 98°C for 30 s, followed by 35 cycles of 98°C for 10 s, 55°C for 20 s, 72°C for 1 min, and a final extension at 72°C for 5 min. Products were either direct Sanger sequenced using the primers detailed in Table S4 or cloned using the Strataclone Blunt PCR Cloning kit (Stratagene) and sequenced using T3/T7 primers. Variant calling was carried out in Geneious version 9 (Biomatters), and phylogeny performed in MEGA version six [13]. Expression of *CYP6ER1* variants was initially assessed by PCR amplification of the *CYP6ER1* coding sequence followed by cloning and sequencing of 16 colonies per strain (as detailed above). Quantitative PCR analysis of the expression of different *CYP6ER1* variants was performed using the primers detailed in Table S4. PCR reactions (20 μl) contained 10 ng of cDNA, 10 μl of SYBR Green JumpStart Taq Readymix (Sigma), and 0.25 μm of each primer. Samples were run on a Rotor-Gene 6000 (Corbett Research) using temperature cycling conditions of: 2 min at 95°C followed by 40 cycles of 95°C for 15 s, 57°C for 15 s and 72°C for 20 s. A final melt-curve step was included post-PCR (ramping from 72°C–95°C by 1°C every 5 s) to check for nonspecific amplification. The efficiency of PCR for each primer pair was assessed using a serial dilution of 100 ng to 0.01 ng of cDNA. Each quantitative RT-PCR experiment consisted of three independent biological replicates with two technical replicates for each. Data were analyzed in Microsoft Excel according to the ΔC_T method [14], using the geometric mean of two reference genes (actin and α2-tubulin) for normalization.

Heterologous expression of P450s

Natural and mutated *CYP6ER1* variants and *M. domestica* NADPH-dependent cytochrome P450 reductase (CPR) (GenBank accession number Q07994) were codon optimized for expression in lepidopteran cells and obtained by gene synthesis (Geneart, CA, USA) in the pDEST8 expression vector (Invitrogen). The PFastbac1 vector with no insert DNA was used to produce a control virus. The recombinant baculovirus DNA was constructed and transfected into Sf9 cells using the Bac-to-Bac baculovirus expression system (Invitrogen) according to the manufacturer's instructions. The titer of the recombinant virus was determined following the protocols of the supplier. High Five cells grown to a density of 2×10^6 cells/mL^{-1} were co-infected with recombinant baculoviruses containing P450 and CPR at various MOI (multiplicity of infection) ratios to identify the best conditions. Control cells were co-infected with the baculovirus containing vector with no insert (ctrl-virus) and the recombinant baculovirus expressing CPR using the same MOI ratios. Ferric citrate and δ-aminolevulinic acid hydrochloride were added to a final concentration of 0.1 mM at the time of infection and 24 h after infection to compensate the low levels of endogenous heme in the insect cells. After 60 h, cells were harvested by centrifugation, washed with PBS, and microsomes of the membrane fraction prepared according to standard procedures [15]. Briefly, pellets were homogenized for 30 s in 0.1M Na/K-phosphate buffer, pH 7.4 containing 1mM EDTA and DTT and 200mM sucrose using a Fastprep (MP Biomedicals), filtered through miracloth and centrifuged for 10 min at 680 g at 4°C. The supernantant was then centrifuged for 1 h at 100,000 g at 4°C, with the pellet subsequently resuspended in 0.1M Na/K-phosphate buffer, pH 7.6 containing 1mM EDTA and DTT and 10% glycerol using a Dounce tissue grinder. P450 expression and functionality was estimated by measuring CO-difference spectra in reduced samples using a dual beam Cary 300 UV-Vis Spectrophotometer (Agilent) and scanning from 500 nm to 400 nm [15]. The protein content of samples was determined using Bradford reagent (Sigma) and bovine serum albumin as a reference following the manufacturer's instructions.

Metabolism assays and LC-MS/MS analysis

Metabolism of imidacloprid was assayed by incubating recombinant P450/CPR (2 pmol P450 / assay) or ctrl-virus/CPR microsomes in 0.1 M potassium phosphate buffer pH 7.6 with an NADPH-regenerating system (Promega; 1.3 mM NADP+, 3.3 mM glucose-6-phosphate, 3.3 mM $MgCl_2$, 0.4 U/mL^{-1} glucose-6-phosphate dehydrogenase) and substrate (12.5 μM) at 27°C for 1 h. The total assay volume was 200 μL using three replicates for each data point. Microsomes incubated without NADPH served as a control. The assay was quenched by the addition of ice-cold acetonitrile (to 80% final concentration), centrifuged for 10 min at 3000 g and the supernatant subsequently analyzed by liquid chromatography-tandem mass spectrometry. Chromatography was performed using a Waters UPLC utilizing a Waters Acquity HSS T3 (2.1x50 mm, 1.8μm) column. Solvents were water/0.1% formic acid and acetonitrile/0.1% formic acid used in a 4 min gradient. The mass spectrometer used was a Sciex API4000 in positive ionization mode for imidacloprid and its hydroxylated metabolite (MRM transitions: 256 > 175, 272 > 191, respectively). 6-CNA was measured in negative ion mode using the ion transition 165 > 121. Quantification was performed by external calibration using reference compounds. Recovery rates of parent compounds using microsomal fractions without NADPH were close to 100%.

Homology modeling of CYP6ER1 and molecular docking simulation

3D models for CYP6ER1vA, CYP6ER1vB and CYP6ER1vL were generated by the advanced homology modeling tool within the Schrodinger software suite [16]. Following a BLAST search for the most suitable template-fold all three protein models were constructed based on the crystal structure of human CYP3A4 (PDB-ID: 1TQN) and refined by an energy minimization step to remove conformational strains and contacts. For the docking studies a catalytic oxygen atom was manually added to the heme iron center. Imidacloprid was docked into the three CYP6ER1 variants using the virtual screening software package LeadIT 2.2.0 utilizing the FlexPharm option [17]. The pharmacophore constraint [18] required a non-hydrogen-atom within a distance of 2.5 Å from the catalytic oxygen. All resulting docking poses underwent hierarchical clustering using a script based on pairwise in-place RMSD values; for this only non-hydrogen atoms were taken into account. The cut height for the cluster generation was 2.0. For each resulting cluster the pose with the lowest FlexX docking rank was selected. This subset of cluster representatives was further reduced by removing all poses where not at least one of the five membered ring atoms was within a distance of 2.5 Å (or less) from the catalytic oxygen. For the models of vL, vA and vB the resulting pose spaces were then visually inspected and compared.

Transgenic expression of candidate genes in *D. melanogaster*

Wild-type (*CYP6ER1vL*) and mutant *CYP6ER1* variants were synthesized and provided in the pUASTattB40 plasmid (Geneart, CA, USA). Using the PhiC31 system, clones were transformed into the germline of a *D. melanogaster* strain carrying the attP40 docking site on chromosome 2 ["y^1w^{67c23}; P attP40 25C6," "1;2"]. The transgenic lines obtained were balanced and the integration of genes confirmed by PCR and sequencing using Phusion DNA polymerase (Thermo) as detailed above with the primers detailed in Table S4. Virgin females of the Act5C-GAL4 strain were crossed with UAS-gene-of-interest males. Bioassays were used to assess the susceptibility of adult female flies to imidacloprid. Several concentrations were overlaid onto 1.5% agar containing 1% sucrose in standard *Drosophila* vials and allowed to dry overnight at room temperature. 10-15 adult flies (two to five days post eclosion) were then added to each vial and mortality assessed after 48 hr. Four replicates were carried out for each concentration. Control mortality was assessed using vials containing agar/sucrose minus insecticide. Lethal concentrations (LC_{50} values) and 95% fiducial limits were calculated by probit analysis using Genstat version 16 (VSN International).

Copy number analysis and sequencing of individual hoppers

Genomic DNA was extracted from multiple pools of 10 hoppers using the Plant DNeasy Mini kit (QIAGEN) and used to construct PCR-free libraries. Libraries of each strain were sequenced across a single lane of an Illumina HiSeq2500 using a 250 bp paired-end read metric. FastQC was used to check the quality of the raw reads obtained [19] and reads trimmed using Trim Galore [20]. In initial attempts to estimate gene copy number the reads of each strain were mapped to the reference genome (sequenced from an inbred line derived from a strain collected in Hangzhou, China, in 2008 [10], GenBank assembly accession number: GCA_000757685.1) using BWA-MEM [21] and CNV estimated using CNVseq [22] with data of each field strain compared to the lab susceptible strain. This analysis failed to identify significant differences in copy number between the strains at the CYP6ER1 locus, likely because the single scaffold of the reference genome where CYP6ER1 is located (KN153994.1) fails to accurately represent the genetic diversity observed at this locus in different variants and strains resulting in, at best, only partial mapping of *CYP6ER1*-related reads. Thus, in a second approach reads were mapped to the coding sequence of *CYP6ER1* and two reference genes: the P450 *CYP6AY1* and the voltage-gated sodium channel (*VGSC*), both of which are single copy genes in BPH, using BWA-MEM [21]. Read coverage was then compared in 100bp non-overlapping windows across the coding sequence of the three genes using SAMtools [23]. Results were validated with qPCR using DNA as template and two sets of primers (designed in conserved regions (variant non-specific) of the gene) for *CYP6ER1* listed in Table S4 and the conditions described above. Data were analyzed according to the ΔΔCT method [24] using the *VGSC* as a reference gene for normalization with the expression values of the four biological replicates obtained for each of the two *CYP6ER1* primer sets averaged.

For variant analysis on individual hoppers, DNA and RNA was extracted (as above) from individuals of the NLF2 and NLF7 strains and used as template in PCR using Phusion DNA polymerase (Thermo) (as detailed above) and the primers detailed in Table S4 which amplify a region containing key SNPs that are diagnostic for different *CYP6ER1* variants.

Gene capture

Approximately 2.5 ug of gDNA was extracted per strain from several pools of insects as detailed above and sent to Earlham Institute for processing according to Roche's NimbleGen gene capture protocol. The gDNA was sheared with a Covaris tube and size selected within the range of 5-9 kb on a BluePippin. The pre-capture library was amplified for 6 rounds to increase the starting material for the capture set up. The library was incubated with the baits (fifteen 100 nt baits per transcript) designed to cover the entire 1.5 kb coding sequence of all known *CYP6ER1* variants described in this study) at 47°C for 22 hr. The post capture library was generated through 19 cycles of amplification of the bait extracted fragments. Each library was sequenced on a single SMRT cell (P6C4). To minimize sequencing errors inherent in PacBio data only circular consensus (CCS) reads, generated from repeated passes of polymerase over a single molecule, or raw reads that were error corrected using the Illumina reads for NLS, NLF2 and NLF7 were used to assemble gene sequences. Assembly and bioinformatics analyses was conducted in Geneious version 9 (Biomatters). Error correction of long Pacbio reads with short-read Illumina data was performed using proovread [25].

Reporter gene assays

Promoter sequences were synthesized, subcloned into pGL3-Basic (Promega) and transformed into Library Efficiency DH5α Competent Cells (Invitrogen). Plasmids were extracted with the GeneJet plasmid miniprep kit (Fermentas), sequenced and then adjusted to 400 ng/μl for use in dual luciferase assays using the Sf9 insect cell line. Approximately 1×10^6 cells per well were plated into 6-well plates 2 hr prior to transfection and allowed to reach 60%–70% confluency. Insect GeneJuice Transfection Reagent (Novagen) was used for transfection of constructs and the Dual-Luciferase Reporter Assay (Promega) used for promoter activity measurements according to the manufacturer's protocols. 2 μg of reporter constructs and pGL3 without insert (as a control) was co-transfected with 4 ng Renilla luciferase pRL-CMV using GeneJuice and incubated at 27°C. 4 hr post-transfection, the transfection mixture was removed and replaced with supplemented Grace's Insect Medium (GIBCO). Following further incubation at 27°C for 48 h and washing of cells with PBS, cells from each well were harvested in 500 μL passive lysis buffer (Promega) and luciferase activity measured on a GloMax 20/20 (Promega). Construct luciferase activity was normalized to Renilla luciferase activity as instructed in the manufacturer's protocol.

QUANTIFICATION AND STATISTICAL ANALYSIS

All statistical analyses were performed in GraphPad Prism 7 (GraphPad Software). Significant differences in expression or copy number in all QPCR experiments were determined using one-way ANOVA with post hoc Tukey HSD. Significant differences in the number of colonies obtained for each variant for each strain in the cloning and sequencing of individuals was determined using a paired t test. Significant differences in normalized reporter gene expression between promoter variants was determined using one-way ANOVA with post hoc Tukey HSD. Statistical details of experiments (value of n, precision measures and definitions of significance) are provided in figure legends.

DATA AND SOFTWARE AVAILABILITY

The sequences reported in this paper have been deposited in the GenBank database (accession numbers GenBank: MF970458, GenBank: MF970459, GenBank: MF970460, GenBank: MF970461, GenBank: MF970462, and GenBank: MF970463).

Current Biology, Volume *28*

Supplemental Information

Neofunctionalization of Duplicated P450 Genes Drives the Evolution of Insecticide Resistance in the Brown Planthopper

Christoph T. Zimmer, William T. Garrood, Kumar Saurabh Singh, Emma Randall, Bettina Lueke, Oliver Gutbrod, Svend Matthiesen, Maxie Kohler, Ralf Nauen, T.G. Emyr Davies, and Chris Bass

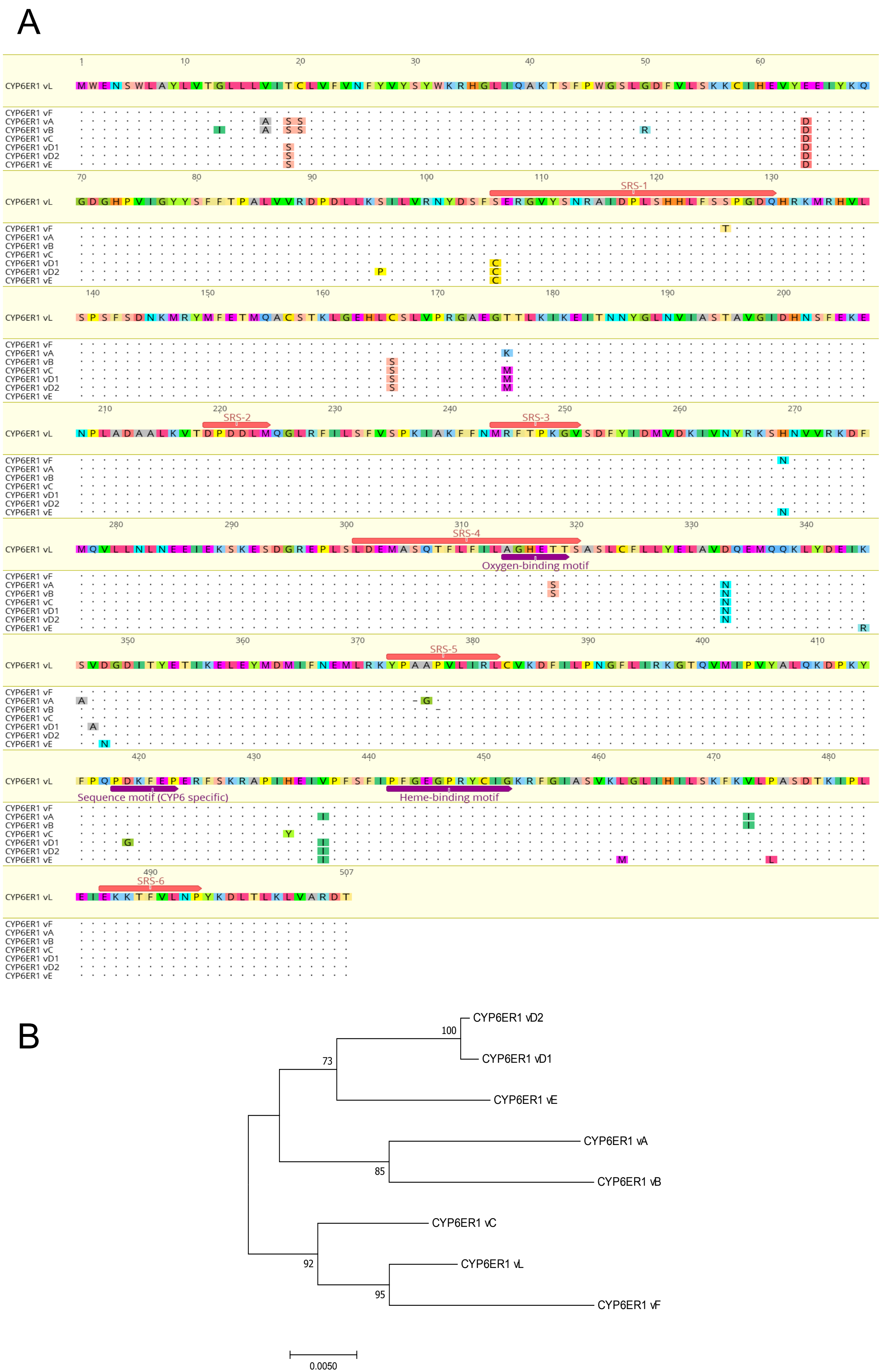
A
CYP6ER1 vL
CYP6ER1 vF
CYP6ER1 vA
CYP6ER1 vB
CYP6ER1 vC
CYP6ER1 vD1
CYP6ER1 vD2
CYP6ER1 vE
SRS-1
SRS-2
SRS-3
SRS-4
Oxygen-binding motif
SRS-5
Sequence motif (CYP6 specific)
Heme-binding motif
SRS-6
B
CYP6ER1 vD2
100
CYP6ER1 vD1
73
CYP6ER1 vE
CYP6ER1 vA
85
CYP6ER1 vB
CYP6ER1 vC
92
CYP6ER1 vL
95
CYP6ER1 vF
0.0050

Figure S1. Sequence characterisation of CYP6ER1 variants. Related to Figure 1.
A) Amino acid alignment of CYP6ER1 variants. Key conserved P450 motifs and substrate recognition sites are annotated. B) Phylogenetic relationship of CYP6ER1 variants. Tree was generated using the Maximum Likelihood method and is drawn to scale, with branch lengths measured in the number of substitutions per site. Bootstrap replications were performed and the percentage of 1000 replications supporting each branch are shown.

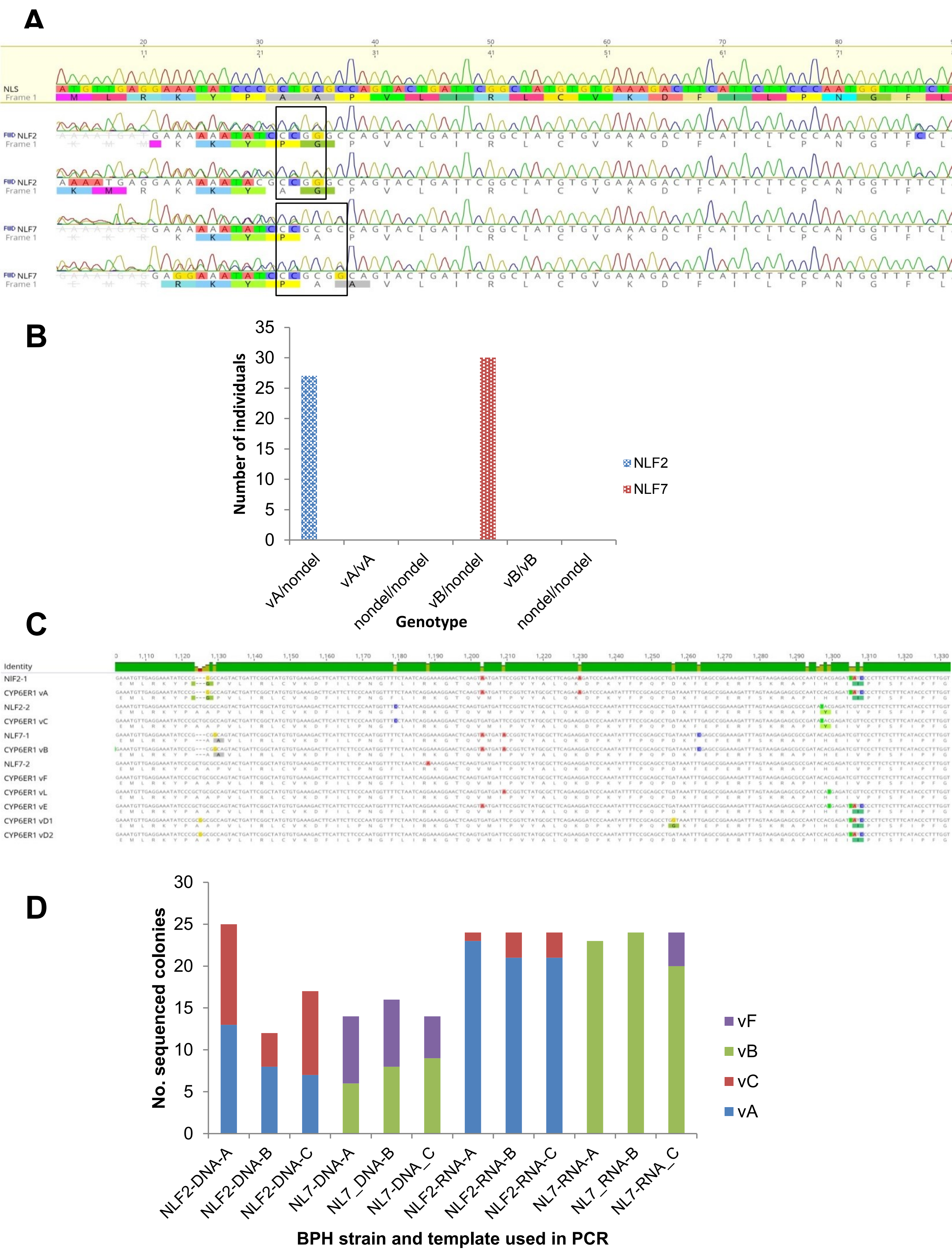

A
NLS
Frame 1
NLF2
Frame 1
NLF2
Frame 1
NLF7
Frame 1
NLF7
Frame 1
B
Number of individuals
NLF2
NLF7
vA/nondel
vA/vA
nondel/nondel
vB/nondel
vB/vB
nondel/nondel
Genotype
C
Identity
NIF2-1
CYP6ER1 vA
NLF2-2
CYP6ER1 vC
NLF7-1
CYP6ER1 vB
NLF7-2
CYP6ER1 vF
CYP6ER1 vL
CYP6ER1 vE
CYP6ER1 vD1
CYP6ER1 vD2
D
No. sequenced colonies
vF
vB
vC
vA
NLF2-DNA-A
NLF2-DNA-B
NLF2-DNA-C
NL7-DNA-A
NL7_DNA-B
NL7-DNA_C
NLF2-RNA-A
NLF2-RNA-B
NLF2-RNA-C
NL7-RNA-A
NL7_RNA-B
NL7-RNA_C
BPH strain and template used in PCR

Figure S2. *CYP6ER1* genotyping of individuals of the NLF2 and NLF7 BPH strains using DNA and RNA as template in (RT-)PCR. Related to Figure 3. (A) Representative sequence traces obtained from direct sequencing of a diagnostic fragment of *CYP6ER1* encompassing the resistance mutation sites in exon 6. DNA extracted from individuals of the NLF2 and NLF7 strains was used as template in PCR. Two representative sequences of each strain are aligned to a representative sequence obtained from NLS (which has just one copy of *CYP6ER1*). Boxed regions indicate the sites of the A375del+A376G mutations in *CYP6ER1v*A and P377del in *CYP6ER1vB* (see also part C of this figure) and highlight the heterozygosity observed at these regions (overlapping chromatogram peaks). (B) Results of genotyping 27-30 individuals of the NLF2 and NLF7 for the resistance mutations that define *CYP6ER1v*A and *CYP6ER1vB*. (C) Alignment of a diagnostic *CYP6ER1* sequence fragment from BPH individuals of resistant strains. Alignment shows representative sequence reads obtained from cloning and sequencing an amplicon containing SNPs and INDELs diagnostic for each of the unique *CYP6ER1* variants using DNA extracted from individuals of the NLF2 and NLF7 strains. For reference the sequence of the eight *CYP6ER1* variants is included. (D) Number of sequenced colonies obtained of each *CYP6ER1* variant after cloning and sequencing PCR products amplified from either genomic DNA or mRNA of individuals of the NLF2 and NLF7 strains.

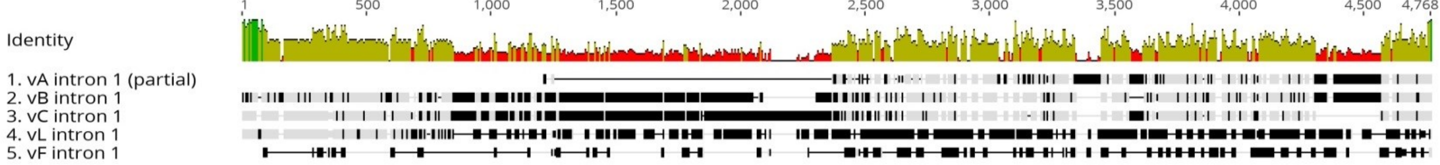

	vA intron 1 (...	vB intron 1	vC intron 1	vL intron 1	vF intron 1
vA intron 1 (partial)		62.788%	53.471%	29.240%	19.647%
vB intron 1	62.788%		83.395%	44.544%	15.689%
vC intron 1	53.471%	83.395%		44.869%	15.148%
vL intron 1	29.240%	44.544%	44.869%		18.416%
vF intron 1	19.647%	15.689%	15.148%	18.416%	

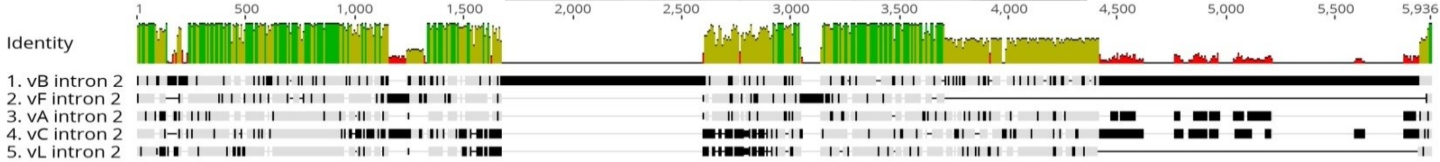

	vB intron 2	vF intron 2	vA intron 2	vC intron 2	vL intron 2
vB intron 2		40.450%	57.032%	53.548%	49.855%
vF intron 2	40.450%		59.553%	56.943%	62.544%
vA intron 2	57.032%	59.553%		74.154%	80.210%
vC intron 2	53.548%	56.943%	74.154%		75.513%
vL intron 2	49.855%	62.544%	80.210%	75.513%	

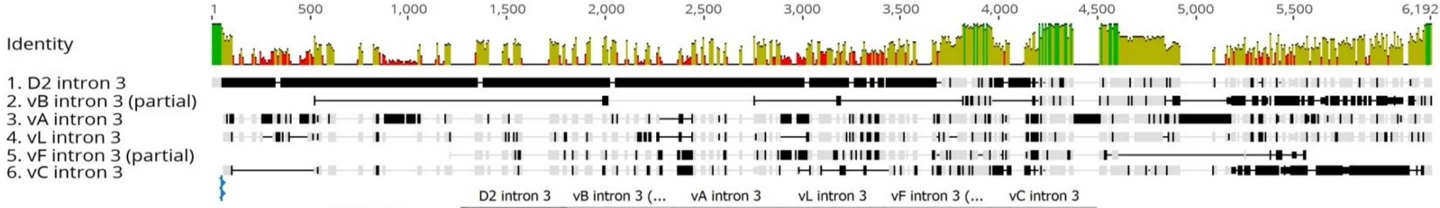

	D2 intron 3	vB intron 3 (...	vA intron 3	vL intron 3	vF intron 3 (...	vC intron 3
D2 intron 3		21.895%	43.938%	44.345%	32.276%	35.004%
vB intron 3 (partial)	21.895%		28.853%	34.383%	18.323%	30.205%
vA intron 3	43.938%	28.853%		78.910%	62.323%	55.845%
vL intron 3	44.345%	34.383%	78.910%		64.527%	63.801%
vF intron 3 (partial)	32.276%	18.323%	62.323%	64.527%		56.042%
vC intron 3	35.004%	30.205%	55.845%	63.801%	56.042%	

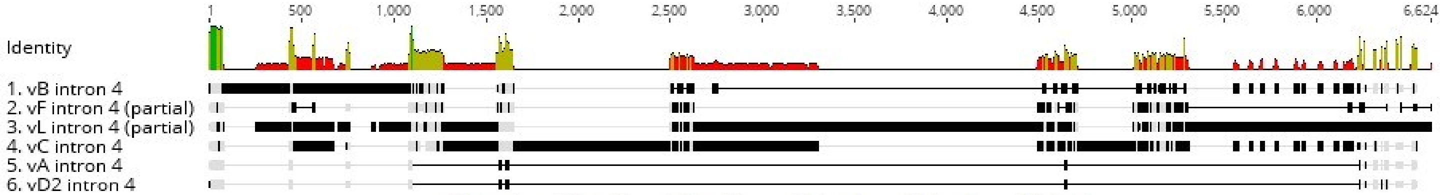

	vB intron 4	vF intron 4 (...	vL intron 4 (...	vC intron 4	vA intron 4	vD2 intron 4
vB intron 4		27.966%	26.264%	25.910%	12.677%	12.540%
vF intron 4 (partial)	27.966%		19.464%	27.521%	18.982%	18.809%
vL intron 4 (partial)	26.264%	19.464%		37.179%	5.025%	4.965%
vC intron 4	25.910%	27.521%	37.179%		7.872%	7.792%
vA intron 4	12.677%	18.982%	5.025%	7.872%		99.051%
vD2 intron 4	12.540%	18.809%	4.965%	7.792%	99.051%	

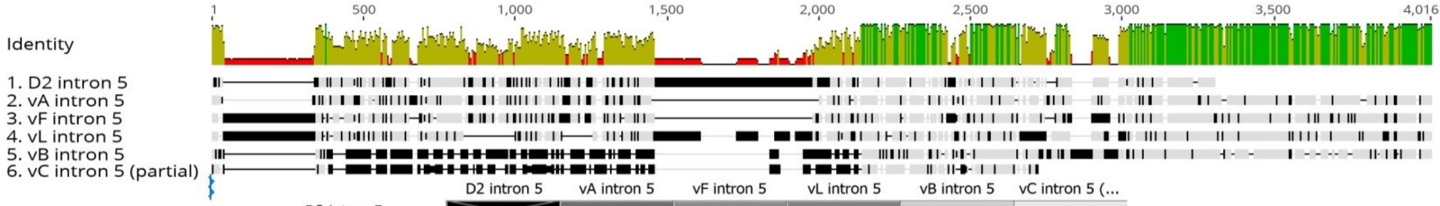

	D2 intron 5	vA intron 5	vF intron 5	vL intron 5	vB intron 5	vC intron 5 (...
D2 intron 5		72.941%	66.422%	68.793%	50.169%	43.824%
vA intron 5	72.941%		81.403%	69.075%	67.483%	54.206%
vF intron 5	66.422%	81.403%		76.612%	60.167%	46.750%
vL intron 5	68.793%	69.075%	76.612%		54.709%	38.457%
vB intron 5	50.169%	67.483%	60.167%	54.709%		91.983%
vC intron 5 (partial)	43.824%	54.206%	46.750%	38.457%	91.983%	

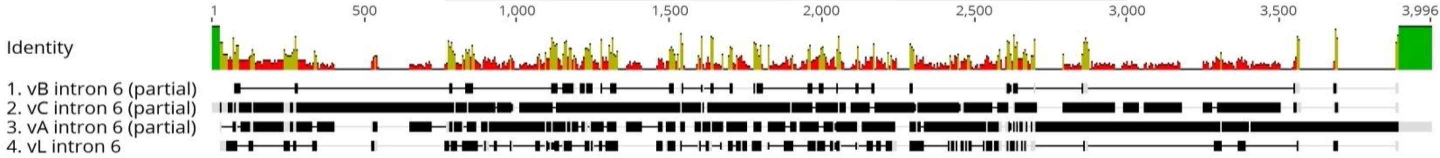

	vB intron 6 (...	vC intron 6 (...	vA intron 6 (...	vL intron 6
vB intron 6 (partial)		7.910%	8.061%	19.212%
vC intron 6 (partial)	7.910%		33.069%	19.281%
vA intron 6 (partial)	8.061%	33.069%		21.562%
vL intron 6	19.212%	19.281%	21.562%	

Figure S3. **Sequence analysis of the introns of different *CYP6ER1* variants. Related to Figure 3.**

Alignment of each intron is shown with a matrix of calculated sequence identity shown below.

A

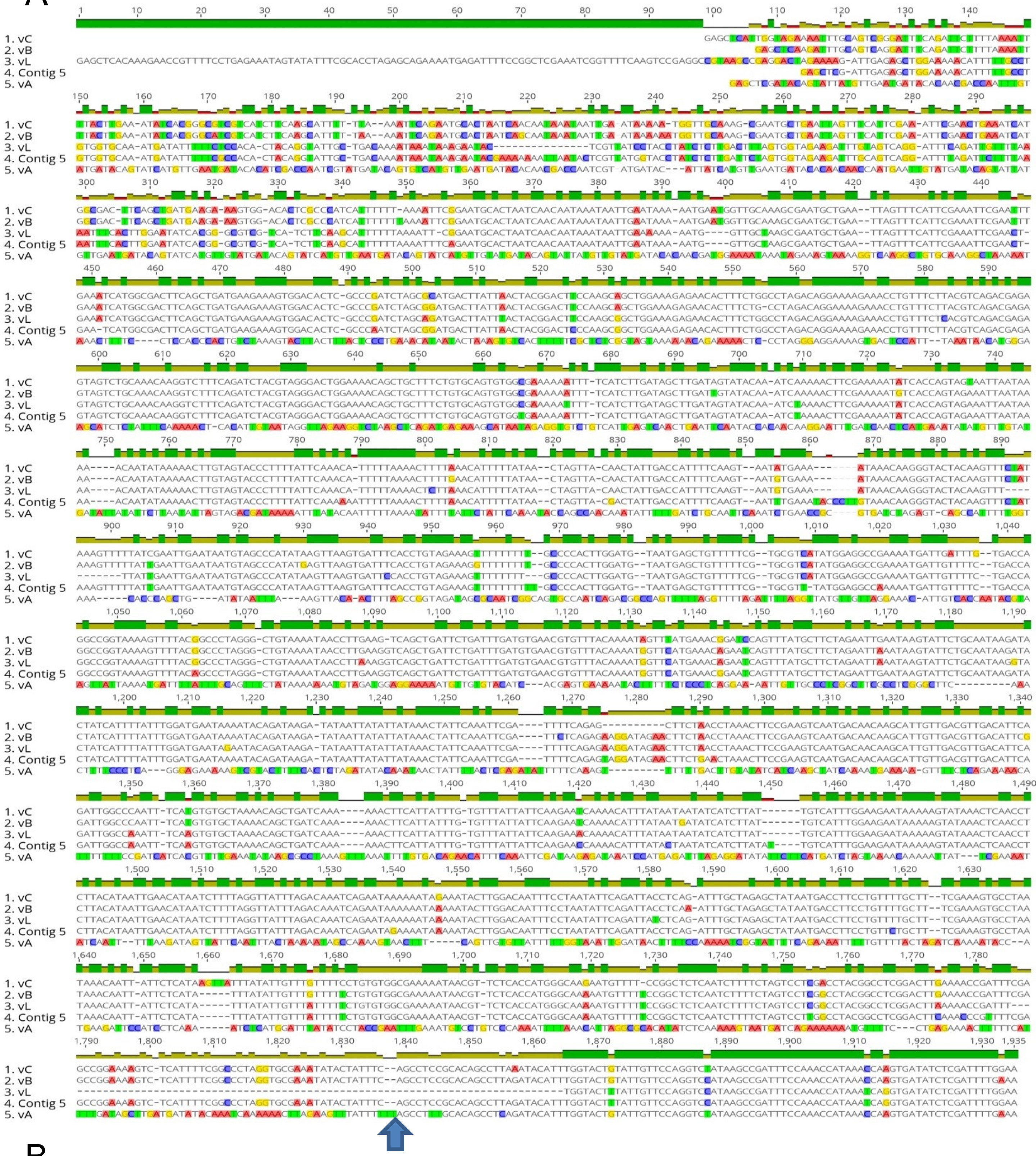

B

	vC	vB	vL	Contig 5	vA
vC		96.708%	82.986%	88.199%	45.985%
vB	96.708%		84.847%	89.128%	45.985%
vL	82.986%	84.847%		90.557%	43.022%
Contig 5	88.199%	89.128%	90.557%		46.340%
vA	45.985%	45.985%	43.022%	46.340%	

Figure S4. **Alignment of *CYP6ER1* promoter variants. Related to Figure 3.** A) Alignment of 1750bp of the putative promoter region upstream of different *CYP6ER1* variants. Contig 5 could not be assigned to a known *CYP6ER1* variant. The position of the sequence breakpoint upstream of *CYP6ER1vA* is illustrated with an arrow. B) Matrix of calculated sequence identity of the different promoter variants.

Name	Year collected	Country of origin	Region/area
**NLS**	1984	Japan	Unknown
**NLF1**	August 2009	Thailand	Unknown
**NLF2**	November 2010	Vietnam	Trà Vinh Province, Southern Vietnam
**NLF3**	August 2011	Vietnam	Hau Giang
**NLF4**	August 2011	Indonesia	Anjatan District, Indramayu
**NLF5**	September 2011	India	Raipur, Chhattisgarth
**NLF6**	March 2012	India	Koppal District, Karnataka State
**NLF7**	April 2012	India	East Godavari District, Andhra Pradesh
**NLF8**	September 2012	India	Sidhikerra, Karnataka State

Table S1. Origin of BPH strains used in this study. Related to Figure 1.

Strain	LC50-value [mg/L^{-1}]	95% limits	Slope (+/- SD)	RR	Mortality 4 mg L^{-1}	Mortality 20 mg L^{-1}
**NLS**	0.6	0.50-0.70	1.822+/- 0.158	1	-	-
**NLF1**	111.7	66.05-212	0.891+/- 0.11	186.2	-	-
**NLF2**	170.2	46.44-5095	0.655+/- 0.221	283.7	-	-
**NLF3**	>1000	-	-	1666	-	-
**NLF4**	-	-	-	-	16.50 (+/-8.42)	20.20 (+/-4.21)
**NLF5**	-	-	-	-	30.14 (+/-8.11)	34.25 (+/-8.14)
**NLF6**	-	-	-	-	15.13 (+/-5.67)	58.31 (+/-8.00)
**NLF7**	14.49	4.55-30.81	0.653+/- 0.134	24.2	-	-
**NLF8**	-	-	-	-	15.74 (+/-5.69)	42.42 (+/-8.02)

Table S2. Sensitivity of susceptible and resistant strains of *Nilaparvata lugens* to imidacloprid topically applied to adult females. **Related to Figure 1**.
Data for strain NLS, NLF1, 2, 3, and 7 shows lethal concentrations 50% (LC_{50} values) determined by probit analyses. Data for NL4, 5, 6, and 8 shows percentage mortality (± standard error) at two diagnostic doses (LD_{95} and 5XLD_{95} of the susceptible strain).

Oligo	Sequence	Purpose
ER1 CDS F1	ATGTGGGAAAACTCGTGGTTGG	PCR/sequencing of CYP6ER1 coding sequence
ER1 CDS F2	GGTTGGCCTAYCTTGTCACAGG	PCR/sequencing of CYP6ER1 coding sequence
ER1 CDS R1	CTAAGTATCTCTCGCTACCAGC	PCR/sequencing of CYP6ER1 coding sequence
ER1 CDS R2	GCTACCAGCTTCAGTGTGAGG	PCR/sequencing of CYP6ER1 coding sequence
CYP6ER1 vL/vF F	CATCCATGAGGTCTACGAAG	CYP6ER1 variant-specific QPCR
CYP6ER1 vL/vF R	GAGTGCTGAACAGATGGTGT	CYP6ER1 variant-specific QPCR
CYP6ER1 vA F	CTTTCTTCACCCCCGCCC	CYP6ER1 variant-specific QPCR
CYP6ER1 vA R	CCTGCATGGTCTCGAACATG	CYP6ER1 variant-specific QPCR
CYP6ER1 vB F	TCTTGTCACAATCCTGTTGCTG	CYP6ER1 variant-specific QPCR
CYP6ER1 vB R	TGGATGCATTTCTTGGACAATACG	CYP6ER1 variant-specific QPCR
CYP6ER1 vC F	GAGACTACTTCTGCATCTTTGT	CYP6ER1 variant-specific QPCR
CYP6ER1 vC R	GGAAACCATTGGGAAGAATGA	CYP6ER1 variant-specific QPCR
CYP6ER1 vD F	AGATCAAATCGGCGGATGGA	CYP6ER1 variant-specific QPCR
CYP6ER1 vD R	CGGAATCATCACTTGAGTTCC	CYP6ER1 variant-specific QPCR
CYP6ER1 vE R	CCGGAATCATTACTTGAGTTCC	CYP6ER1 variant-specific QPCR
CYP6ER1 vE F	GTATGATGAGATCAGATCTGTGA	CYP6ER1 variant-specific QPCR
D099 pUAST F	TCACTGGAACTAGGCTAGCA	Sequence validation of transgenic flies
D102 pUAST F	GGATCCAAGCTTGCATGCCTG	Sequence validation of transgenic flies
D100 pUAST R	AAAGGCATTCCACCACTGCT	Sequence validation of transgenic flies
D101 pUAST R	CCACCACTGCTCCCATTCAT	Sequence validation of transgenic flies
ER1 deletion F	GCAGAAATGTTGAGGAAATATCCC	Sequencing of exon 6 of BPH individuals
ER1 deletion R1	ACCAAAGGGTATGAAAGAGAAGG	Sequencing of exon 6 of BPH individuals
Ex3 qPCR F2	GAATGTGATTGCCTCCACGG	Copy number QPCR of CYP6ER1
Ex3 qPCR R2	AGCATCAGCAAGTGGGTTCT	Copy number QPCR of CYP6ER1
Ex4 qPCR F2	AACATGAGGTTCACGCCGAA	Copy number QPCR of CYP6ER1
Ex4 qPCR R2	TGCATGAAATCCTTCCTCACCA	Copy number QPCR of CYP6ER1
VGSC qPCR F2	CACCATTGTCACACAGCAGC	qPCR of reference gene (VGSC)
VGSC qPCR R2	CCCTGGAGTAGTGCTTGTCG	qPCR of reference gene (VGSC)
Nl_Actin_F	TAACGAGAGGTTCCGTTGCC	qPCR of reference gene (actin)
Nl_Actin_R	GACAGGACAGTGTTGGCGTA	qPCR of reference gene (actin)
Nl_α2_tubulin_F	CCACCCTGGAACACTCTGAC	qPCR of reference gene (α2_tubulin)
Nl_α2_tubulin_R	CGAAGCAGTGATCGAGGACA	qPCR of reference gene (α2_tubulin)

Table S3. Sequence of oligonucleotide primers used in this study. **Related to STAR methods**.
